# Sustainable biohydrogen production from banana peels using microbial fermentation

**DOI:** 10.1186/s12896-025-01080-3

**Published:** 2025-12-13

**Authors:** Mohamed Hemida Abd-Alla, Shymaa Ryhan Bashandy, Wafaa Abdelnaser Sleem, David Mamdouh Khalaf

**Affiliations:** Botany and Microbiology Department, Faculty of Science, Assiut University, Assiut, 71516 Egypt

**Keywords:** Banana peels, Biofuel production, Waste management, Anaerobic digestion, Chicken manure, Hydrogen production, Kinetic analysis, Phenolic compounds

## Abstract

**Background:**

Global energy demand and environmental concerns are driving the search for sustainable alternatives. Banana peels, which account for 30–40% of the 139 million tons of bananas produced annually, are rich in organic matter and offer a promising source for biofuel production. To investigate this potential, experiments were conducted to assess their suitability for biofuel generation.

**Methods:**

Microbial conversion of banana peels into hydrogen and acetone-butanol-ethanol (ABE) was investigated through anaerobic fermentation and enzymatic hydrolysis. Various inocula were tested for anaerobic digestion. Peels concentration kinetics were analyzed, and bacterial isolates were screened for their ability to degrade phenolic compounds, produce cellulase and pectinase, and generate biofuels. The most efficient isolate was identified using 16 S rRNA sequencing.

**Results:**

Findings demonstrate that banana peels have a high volatile solids content of 93.7%, a rich carbohydrate profile (550 mg/g reducing sugars, 133.25 mg/g total carbohydrates), and a balanced C/N ratio of 21.5, making them a promising substrate for biofuel production and waste management. In evaluating inoculum performance, chicken manure proved to be the most effective inoculum, producing 846.6 mL/L of hydrogen with a bacterial count of 12.67 × 10⁵ CFU/mL, followed by cow dung (283.3 mL/L of hydrogen). Soil inoculum did not result in hydrogen production despite microbial activity. Furthermore, the optimal hydrogen production was achieved at a 20% (w/v) banana peels concentration, reaching 1400 mL/L, with higher concentrations (40%) showing inhibition. The Gompertz model confirmed the peak performance at 20% concentration (Hₘₐₓ = 1330 mL, Rₘₐₓ = 130 mL/h, R² = 0.99). Among bacterial isolates, isolate W26 (Bacillus stercoris, 99.93% 16 S rRNA identity) from cow rumen produced the highest hydrogen (1750 mL/L), while W17 excelled in ABE production (1.033 g/L, primarily ethanol). Bacterial isolates W17, W18 and W22 demonstrated cellulase activity, while W13, W20, W23 and W24 exhibited pectinase activity, with W26 showing both. Tolerance to phenolic compounds varied among isolates, with gallic acid, ferulic acid, quercetin, and tannic acid supporting growth in most isolates, unlike pyrogallol. Collectively, these findings highlight the potential of banana peels for sustainable biofuel production, with chicken manure and *Bacillus stercoris* as the optimal inoculum and isolate, respectively.

**Conclusions:**

Based on these findings, banana peels are a promising biofuel substrate due to their high carbohydrate content and favorable C/N ratio. Chicken manure and bacterial isolate W26 (*Bacillus stercoris*) were found to boost hydrogen production at a 20% peels concentration, yielding 1400 mL/L and 1750 mL/L, respectively. Some isolates exhibited cellulase, pectinase, and ABE production capabilities, with W17 achieving the highest ethanol yield of 0.930 g/L. These results highlight the viability of banana peels for eco-friendly bioenergy production and effective waste management.

**Supplementary Information:**

The online version contains supplementary material available at 10.1186/s12896-025-01080-3.

## Introduction

Humanity is facing serious challenges due to energy shortages and environmental degradation. These issues are driven by growing global energy demands and the limited supply of fossil fuels [1]. A critical consequence of this reliance is the significant release of carbon dioxide (CO₂) into the atmosphere. In 2024, fossil fuel combustion produced about 37.4 billion tonnes of CO₂, a 0.8% increase from the previous year. Coal contributed 41%, oil 32%, and gas 21% to these emissions [2]. Land-use changes, such as deforestation, added another 4.2 billion tonnes, raising the total global CO₂ emissions to 41.6 billion tonnes in 2024 [2]. Despite efforts by natural systems to mitigate these emissions, oceans and forests absorbed nearly half of this CO₂. Oceans removed about 26.5%, and land vegetation absorbed around 25% [3]. However, the remaining CO₂ stayed in the atmosphere, causing global temperatures to rise. By 2024, atmospheric CO₂ levels reached 422.5 parts per million, which is 52% higher than pre-industrial levels [2]. Around 3 billion tonnes of CO₂ are released each year without being captured, worsening climate change and harming ecosystems [4].

To address this environmental crisis, renewable energy sources are increasingly recognized as viable alternatives to fossil fuels. One promising method is converting organic waste, especially fruit waste, into energy [5]. Fruit waste includes inedible, spoiled, or discarded parts produced during harvesting, transport, and food processing. It contains high levels of lignocellulosic biomass, making it suitable for bioenergy production. This approach helps meet energy needs while reducing environmental pollution [6].

Among various fruit wastes, banana peels are particularly notable for their abundance and potential. In 2023, the Food and Agriculture Organization (FAO) reported global banana production at 139 million tons, grown on about 6 million hectares. Egypt contributed 1.19 million tons [7, 8]. Banana peels make up 30–40% of the fruit’s weight, resulting in an estimated 42 million tons of peel waste annually. Most of this waste ends up in underutilized landfills [9].

The chemical composition of banana peels supports their use as a bioenergy feedstock. Chemical analysis of dried unripe banana peel powder shows high levels of starch (41%), cellulose (9.3%), hemicellulose (3.2%), pectin (7.4%), lignin (2.3%), and water-soluble reducing sugars (2.1%) [10]. Ripe peels contain up to 30% free sugars and essential minerals, including macroelements like potassium, phosphorus, and magnesium, and microelements such as calcium, iron, zinc, manganese, and copper [11]. Devatkal et al. reported an average phenolic content of 55.02 mg gallic acid equivalent per gram of banana peels [12]. Another study identified quinic acid as the main phenolic compound in both ripe and unripe peels across different cultivars [13].

Given their rich composition, banana peels have been increasingly explored for bioenergy production. Efforts to convert fruit waste into renewable energy have grown steadily. One particularly promising avenue is the production of hydrogen, a versatile fuel for industry and transport. Certain microorganisms produce hydrogen using enzymes called hydrogenases, which either reduce protons or oxidize hydrogen molecules. Microbial fermentation is a major pathway for biohydrogen production [14, 15]. These microbes convert organic matter into CO₂ and hydrogen. The process is classified into dark fermentation, photo fermentation, and combined dark-photo fermentation, depending on light conditions [16].

In dark fermentation, hydrogen is produced during acidogenesis, which breaks down biowaste into fatty acids and other byproducts. These can further convert into hydrogen and acetate through acetogenesis [17]. Recent research has demonstrated the potential of banana peels as a bioenergy feedstock. Housagul et al. produced methane by co-fermenting banana peels with glycerol [18]. Another study used a two-stage anaerobic digestion process to generate both hydrogen and methane [19]. Da Silva Mazareli et al. found that *Bacillus* sp. RM1 produced hydrogen via the acetic/butyric pathway using banana waste [20]. Experiments using Plackett-Burman designs identified headspace volume, temperature, and initial pH as key factors affecting hydrogen yield from banana waste with indigenous inocula [21]. Co-digestion of banana plant waste with sewage sludge has also shown improved biohydrogen and biomethane production [22].

Despite these advances, significant challenges remain in optimizing biohydrogen production from banana peels. One major challenge is the presence of phenolic compounds in banana peels, which can inhibit microbial activity and reduce hydrogen yield. There is limited research on bacterial strains that can tolerate or degrade phenolics to improve fermentation efficiency. The metabolic pathways and mechanisms used by phenolic-tolerant bacteria are also poorly understood.

To address these gaps, this study evaluates the use of banana peels water extracts for hydrogen production. Different peel concentrations and bacterial strains capable of tolerating or degrading phenolic compounds were tested to assess their effectiveness in enhancing biohydrogen production from banana peel waste.

## Materials and methods

### Effect of various inocula on anaerobic digestion of banana peels

#### Inocula collection

**Fig. 1:**
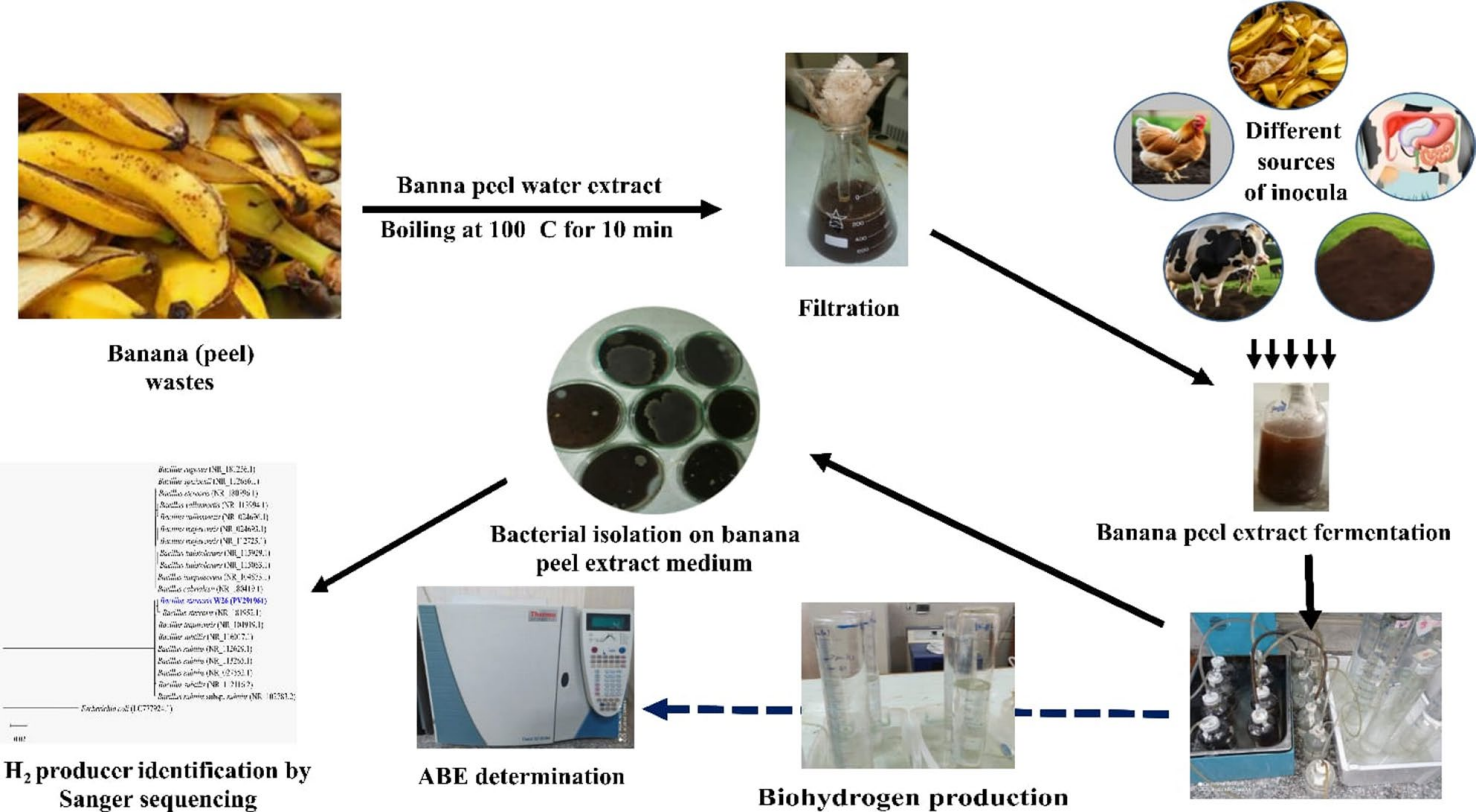
Schematic flowchart of the experimental design used in the study

Five distinct inocula were utilized in batch anaerobic digestion experiments: banana peels (serving as the control), chicken manure, cow rumen fluid, cow dung and soil. Chicken manure was sourced from local animal and poultry farms in the Assiut governorate of Egypt, while soil was collected from nearby agricultural land. Cow rumen fluid was obtained from a slaughterhouse in the Assiut governorate, collected in a 1-liter sterilized glass bottle within 15 min of slaughtering a two-year-old cow. To preserve their integrity, all inocula were stored at 4 °C in a refrigerator until needed. The anaerobic digestion process was initiated by combining the fresh inocula with banana peels, maintaining a consistent inoculum concentration of 10% (w/v) across all trials. The study’s experimental design was illustrated in a schematic flowchart (Fig. 1).

#### Banana peel extract medium preparation

Fresh ripe banana peels, serving as the substrate, were obtained from a local market in the Assiut governorate of Egypt. To prepare the substrate slurry, 200 g of banana peels were mixed with 1 L of tap water. The mixture was homogenized using a household blender and then boiled at 100 °C for 10 min to facilitate breakdown and sterilization. After cooling, the biomass was filtered through a 100-µm muslin cloth to remove coarse solids. The pH of the filtrate was adjusted to 6.8 using 1 M HCl. This process aimed to develop a banana peel extract optimized for hydrogen (H₂) production, with treatments yielding the highest H₂ output selected for subsequent experiments. In this study, an aqueous extract of banana peels was used as a fermentation substrate to control the concentrations of soluble organic compounds, such as sugars and phenolic compounds, important for microbial hydrogen production and inhibition assessment.

#### Batch anaerobic digestion tests with banana peels

Fifteen 600 mL glass flasks were thoroughly cleaned and prepared for batch experiments, with each treatment performed in triplicate. 10% of each tested inoculum (cow dung, chicken manure, cow rumen fluid, and soil) was added to 500 mL of banana peel extract medium and mixed thoroughly until homogenized. To establish anaerobic conditions, each flask was sealed with a rubber stopper fitted with two needle tubes for gas exchange and flushed with nitrogen gas for 2 min, then rapidly sealed with white butyl-rubber sealant [23]. The flasks were incubated in a water bath (YCW-03 S) at 30 °C, and hydrogen (H₂) production was monitored daily until it became negligible [24]. The evolved gas was collected in an inverted cylinder submerged in water and connected to a 2 M NaOH solution to absorb CO₂. Key parameters, such as the total bacterial community count after 24 h and the cumulative hydrogen yield after 48 h, were measured in this study. A 3.0 mL sample was collected every 24 h using a syringe and then centrifuged at 4246 g and 4 °C for 5 min. The supernatant was used for analyzing biofuel products and residual reducing sugars. One microliter of the sample was injected into a gas chromatography apparatus (Thermo Scientific TRACE GC Ultra) with a flame ionization detector connected to a recorder integrator. The resulting supernatant was analyzed via gas chromatography using a Thermo Scientific TRACE GC Ultra system equipped with a flame ionization detector and a recorder-integrator. Separation occurred in a CP-PoraBOND U fused silica PLOT column (25 m × 0.32 mm, df = 7 μm), with injector and detector temperatures set at 250 °C, and oven temperature programmed from 50 °C to 155 °C at a rate of 5 °C per min. Nitrogen served as the carrier gas at a flow rate of 3.5 mL/min [25]. Yield was calculated as the total acetone–butanol–ethanol (ABE) produced divided by the total sugar consumed and expressed in grams per gram.

#### Analytical methods

The banana peel extract samples were analyzed for total solids (TS), volatile solids (VS), alkalinity, pH, carbohydrates, proteins, and chemical oxygen demand (COD) according to standard methods [26]. Additionally, carbon and nitrogen contents were determined using a CHNS analyzer (Analysensysteme GmbH, Donaustr-7, D-63452 Hanau, Germany) to characterize the elemental composition of the banana peel waste.

### Isolation of bacterial communities from anaerobic digestion of banana peels using various inocula

The total bacterial populations involved in the anaerobic digestion of banana peels were quantified using a banana peel extract agar medium. Counts were recorded at 24-hour intervals to track microbial activity over time. The efficacy of each inoculum was evaluated based on its bacterial community density. Following fermentation, surviving hydrogen-producing bacteria were isolated from the spent medium via the streaking method on a modified banana peel extract agar (BPEA) medium. This medium was designed to cultivate and enumerate bacteria capable of degrading banana peels and tolerating their phenolic compounds. The BPEA was prepared using 200 g/L of banana peel extract (as described in Sect. “Banana peel extract medium preparation”), adjusted to pH 6.8, supplemented with 18 g/L agar, and sterilized. The osmotic balance of the banana peel extract agar (BPEA) medium was maintained by the intrinsic mineral content of the banana peel extract. This extract naturally contains essential macro- and micronutrients, including potassium, calcium, magnesium, and phosphorus. No additional salts were supplemented, as preliminary experiments demonstrated sufficient microbial growth and viability under these conditions.

Plates were incubated anaerobically (20% CO₂) at 30 °C for 48 h. Distinct bacterial colonies were selected, purified on fresh BPEA medium under anaerobic conditions, and stained for identification. Purified isolates were preserved on BPEA slants at 4 °C under anaerobic conditions for future use.

### Determining the optimal concentration of banana peels for hydrogen production

To determine the optimal banana peel concentration for maximizing hydrogen production, fermentation trials were conducted using varying substrate concentrations (10%, 20%, 30%, and 40% w/v). Each fermentation vessel contained 500 mL of banana peel extract medium at the specified concentration and was inoculated with 10% (w/v) of chicken manure (550 mL total working volume). The vessels were maintained at 30 °C under anaerobic conditions to prevent oxygen interference. Hydrogen gas was collected in an inverted cylinder submerged in water, and a 2 M NaOH solution was used to remove carbon dioxide from the gas stream. Cumulative hydrogen yields were calculated after 48 h for each concentration, and the results were compared to determining the concentration yielding the highest hydrogen output.

### Kinetics study

The Gompertz model (Eq. 1) was used to analyze hydrogen yields from chicken manure to predict hydrogen production potential (H), maximum production rate (R_max_), and lag phase (λ). Equation 2 is used to calculate coefficient of determination (R²) [27, 28].

1 $$\:H\left(t\right)={H}_{max}.\:exp\left\{-exp\left[\frac{{R}_{max}.e}{{H}_{max}}\left(\lambda\:-t\right)+1\right]\right\}$$

2 $${\mathrm{R}}^2=1-\:\frac{\sum\:\left({H}_{obs}-{H}_{pred}\right){\begin{array}{c}2\\\:\end{array}}^{}}{\sum\:\left({H}_{obs}-{H}_{mean}\right){\begin{array}{c}2\\\:\end{array}}^{}}$$

where:

- H(t) is the cumulative hydrogen production at time t.
- H_*max*_ is the maximum hydrogen production.
- λ\lambda is the lag-phase time.
- The constant e is approximately equal to 2.7.

The Gompertz model was applied to describe the kinetics of cumulative hydrogen production, as it effectively captures the sigmoidal fermentation profile, including lag phase (λ), maximum production rate (Rₘₐₓ), and total hydrogen yield (Hₘₐₓ). This model is widely used in biohydrogen studies due to its reliability in fitting batch fermentation data and evaluating microbial performance under anaerobic conditions.

### Effect of different phenolic compounds on the growth of bacterial isolates

To assess the tolerance of bacterial isolates to phenolic compounds, five compounds (gallic acid, ferulic acid, pyrogallol, quercetin, and tannic acid) were individually incorporated into nutrient agar medium [29] at a final concentration of 1% (w/v). This level was selected based on literature reports indicating that banana peel extracts may contain bioavailable phenolics ranging from 180 to 900 mg/100 g, depending on cultivar and extraction method. Although higher than natural concentrations, this dosage was used to identify robust strains capable of tolerating phenolic stress. Each compound was added to the sterilized medium. Bacterial isolates were inoculated onto the phenolic-supplemented agar plates, which were then incubated at 30 °C for 48 h. Growth was evaluated by observing colony development, with a positive response defined as visible growth exceeding a diameter of 2 mm. This approach allowed the identification of isolates capable of withstanding phenolic stress, a key factor in banana peel degradation. This level was selected based on preliminary trials and literature reports indicating that banana peel extracts may contain bioavailable phenolics ranging from 180 to 900 mg/100 g, depending on cultivar and extraction method. Although higher than natural concentrations, this dosage was used to identify robust strains capable of tolerating phenolic stress.

### Examination of cellulase and pectinase enzyme activity of bacterial isolates

To evaluate the hydrolytic capabilities of bacterial isolates involved in anaerobic digestion of banana peels, enzyme activity assays for cellulase and pectinase were conducted. Although banana peel extract was used as the fermentation substrate, it retained soluble and colloidal fractions of pectin and cellulose-derived compounds. Therefore, evaluating pectinase and cellulase activity was essential to identify bacterial strains capable of degrading these residual components. Such enzymatic traits are also valuable for future applications involving whole peel fermentation, where insoluble lignocellulosic matter is more prevalent. For cellulase activity, isolates were tested on a carboxymethyl cellulose (CMC) agar medium with the following composition (g/L): 10.0 carboxymethyl cellulose, 10.0 peptone, 2.0 (NH₄)₂SO₄, 2.0 K₂HPO₄, 0.3 MgSO₄·7 H₂O, 2.0 gelatin, and 18.0 agar. A simple spot method was employed, where a small drop of each purified bacterial inoculum was placed onto the CMC agar [30]. After incubation at 30 °C for 48 h, plates were flooded with a 0.3% (w/v) congo red solution for 30 min, followed by washing with 1 M NaCl. The presence of a clear hydrolysis zone (halo) around the colonies confirmed cellulolytic activity. For pectinase activity, a Pectinase Screening Agar Medium [31] was prepared with the composition (g/L): 2.0 NaNO₃, 0.5 KCl, 0.5 MgSO₄, 1.0 K₂HPO₄, 0.5 tryptone, 20.0 agar, and 10.0 citrus pectin. Plates were inoculated, incubated at 37 °C for 3 days, and subsequently flooded with 1% aqueous iodine solution to visualize hydrolysis zones. Clear zones around colonies indicated pectin degradation. These assays provided insights into the isolates’ ability to break down complex polysaccharides in banana peels.

### Screening of bacterial isolates for H₂ and biofuel production

Bacterial isolates were first streaked onto fresh banana peel extract agar (BPEA) plates to confirm purity. Their potential for hydrogen (H₂) and biofuel production was then tested using banana peel medium. Inocula were prepared by transferring 1 mL of a 48-hour-old culture into 100 mL of sterilized fluid thioglycolate broth. These bottles were sealed with rubber septa, flushed with nitrogen to ensure anaerobiosis, and incubated at 37 °C with magnetic stirring at 120 rpm for 48 h. Subsequently, 10% (v/v) of this inoculum was injected into 600-mL glass bottles containing 500 mL of banana peel medium, leaving 50 mL as headspace (550 mL total working volume). The bottles were sealed, flushed with nitrogen, and incubated at 30 °C. Evolved gas was collected for 48 h until it stopped in an inverted cylinder submerged in water, connected to a 2 M NaOH solution to absorb CO₂. After 72 h, samples were withdrawn and analyzed for acetone, butanol, ethanol, and butyric acid concentrations using a gas chromatograph (Trace GC Ultra) equipped as described above with a flame ionization detector and recorder integrator. Isolates exhibiting high H₂ and biofuel yields were selected for further characterization.

### Characterization and identification of high H₂-producing isolates

High-performing isolates were characterized morphologically and biochemically. Macroscopic and microscopic observations, including Gram staining, were conducted to assess colony and cellular traits. Biochemical tests growth on MacConkey agar, KOH test, catalase test, starch hydrolysis, gelatin hydrolysis, lipase test, urease test, H₂S production, indole test, and carbohydrate fermentation (glucose, sucrose, lactose, fructose, maltose, mannitol, glycerol, sorbitol) were performed following protocols outlined in Bergey’s Manual [32]. For genotypic identification, DNA was extracted from the most potent H₂-producing isolate following the protocol outlined by Abd-Alla et al. [33]. The 16 S rRNA gene was sequenced by SolGent Co., Ltd. (Bio Industry Development Site, 63 − 10 Hwaan-Dong, Yuseong-Gu, Daejeon, South Korea). Sequences were analyzed using the Basic Local Alignment Search Tool (BLAST) on the NCBI website (http://www.ncbi.nlm.nih.gov/blast/Blast.cgi*).* Sequence editing and assembly were performed with BioEdit version 7.2.5 (http://www.mbio.ncsu.edu/BioEdit/bioedit.html*)*, and BLASTN searches confirmed identity. Phylogenetic trees were constructed using Mega11, incorporating 16 S rRNA sequences of standard bacterial strains from GenBank. The nucleotide sequence of the isolate was deposited in the GenBank database.

### Statistical analysis

The experimental data were analyzed using one-way analysis of variance (ANOVA) with PC-Stat version A software (Copyright 1985, The University of Georgia). Mean comparisons among treatments were performed using the least significant difference (LSD) test at a significance level of 0.05 [34].

## Results

### Chemical characteristics of banana peels

The chemical properties of banana peels, summarized in Table 1, provide valuable insights into their nutritional potential for microbial activity and their suitability for waste management and bioconversion applications. A notably high volatile solids (VS) content of 93.7% indicates a substantial presence of organic matter, making banana peels an excellent candidate for processes such as composting or biogas production. The slightly alkaline nature of the peels, as revealed in Table 1, creates favorable conditions for microbial growth, enhancing their biodegradability. However, the low buffering capacity, measured at 0.2 mg CaCO₃/L, suggests limited resistance to pH fluctuations during microbial processing.

Banana peels exhibit a COD of 40.16 mg/g, reflecting a rich reservoir of organic material that can be harnessed for energy generation. While their protein content is relatively modest at 0.136 mg/g, the peels are abundant in carbohydrates, with reducing sugars amounting to 550 mg/g. This high sugar content underscores their potential as a fermentable substrate for biofuel production. Additionally, Table 1 reports a total carbohydrate content of 133.25 mg/g, encompassing not only reducing sugars but also non-reducing sugars and starches, further amplifying the energy value of the peels.

Elemental analysis reveals a carbon content of 37.03%, alongside moderate levels of nitrogen (1.721%) and hydrogen (3.853%), with a minimal sulfur presence (0.689%). These values yield a carbon-to-nitrogen (C/N) ratio of approximately 21.5, as shown in Table 1. This balanced ratio suggests an adequate supply of carbon to serve as an energy source, paired with sufficient nitrogen to sustain microbial growth and reproduction during bioconversion processes. Collectively, the data in Table 1 highlights the multifaceted potential of banana peels, positioning them as a valuable resource for biofuel production, waste management strategies, and nutrient recovery across various industries.

**Table 1:** Chemical characteristics of banana peel extract

Parameter	Value
TS%	34.98
VS%	93.7
C%	37.03
N%	1.721
H%	3.853
S%	0.689
Protein (mg/g)	0.136
Reducing sugar (mg/g)	550
Carbohydrates (mg/g)	133.25
pH	8.1
Alkalinity (mg CaCO_3_/L)	0.2
COD (mg/g fresh wt)	40.16

### Effect of various inocula on anaerobic digestion of banana peel

**Fig. 2:**
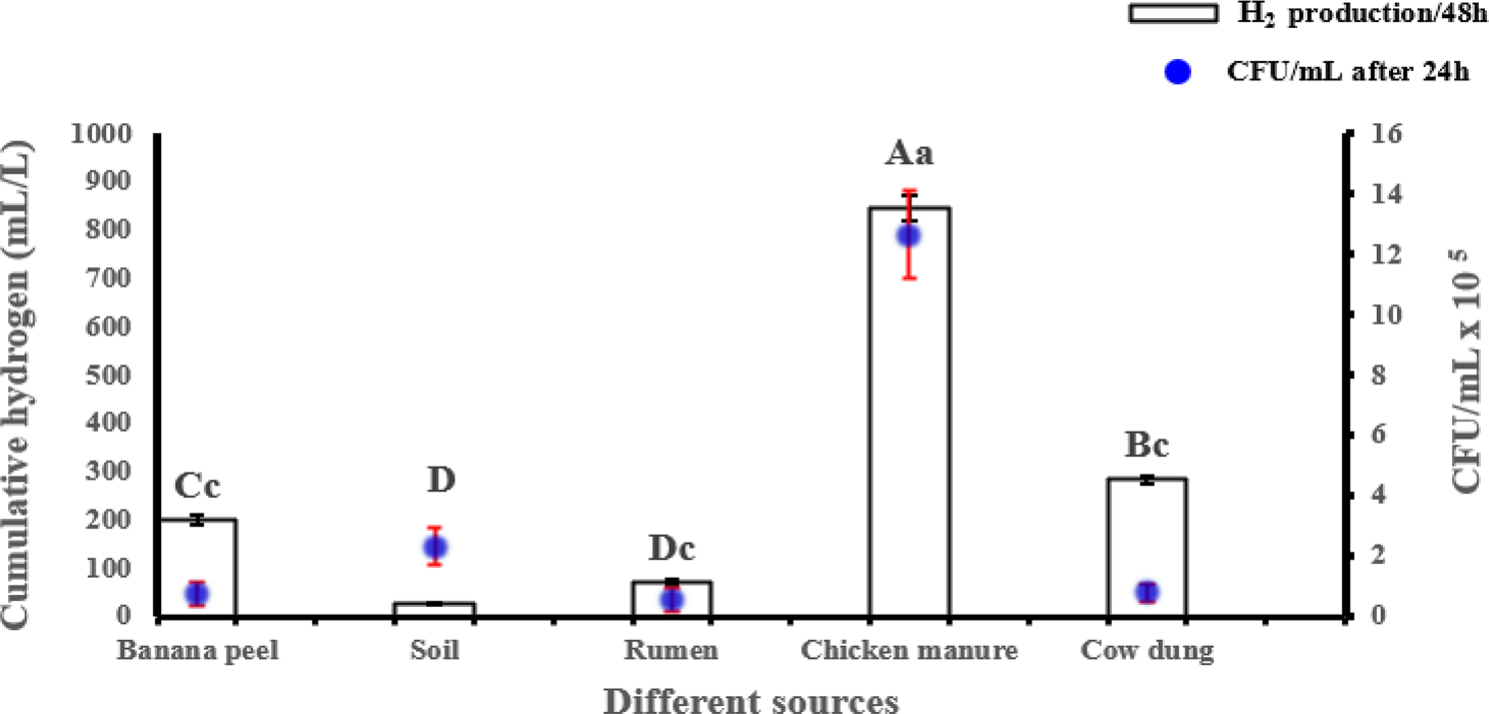
Influence of various inocula on the total bacterial community count after 24 h and hydrogen production after 48 h during the anaerobic digestion of banana peels. Each column represents the mean of three replicates ± SE (indicated by vertical bars). Means with the same capital letters indicate that cumulative hydrogen production from different inoculum sources is not significantly different at the 0.05 level, while means with the same lowercase letters indicate no significant difference in total bacterial community count isolated from different inoculum sources, based on the least significant difference (LSD) test

The results of this investigation reveal distinct differences in the performance of various inocula during the anaerobic digestion of banana peels, as depicted in Fig. 2. Among the tested sources, chicken manure proved significantly the most effective for hydrogen production, achieving a yield of 846.6 mL/L and supporting a robust total bacterial population of 12.67 × 10⁵ CFU/mL. Cow dung followed as the next most productive inoculum, generating 283.3 mL/L of hydrogen with a bacterial count of 8.0 × 10⁴ CFU/mL. In contrast, soil displayed significant microbial activity but failed to produce detectable levels of hydrogen, suggesting its microbial community may not be suited for this specific bioconversion process. Banana peels and cow rumen, meanwhile, exhibited moderate outcomes of hydrogen production. On the other hand, there was no significant difference in the total bacterial count among banana peel, cow rumen, and cow dung inocula. These findings, illustrated in Fig. 2, highlight chicken manure as the standout inoculum for optimizing hydrogen production from banana peel waste.

**Fig. 3:**
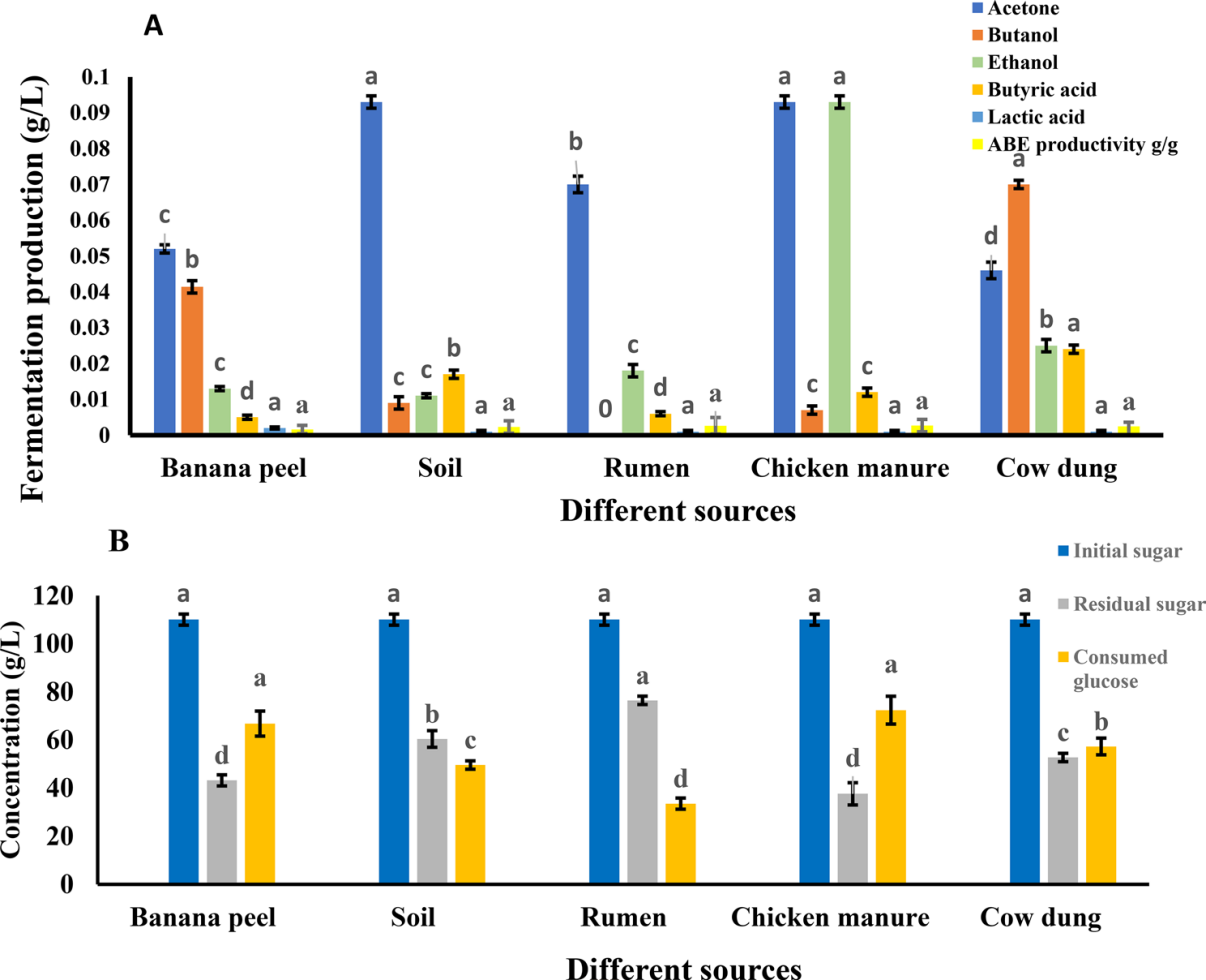
Biofuel products and ABE productivity (**A**), Initial sugar, residual sugar and consumed glucose (**B**) after 48 h from banana peels by anaerobic digestion using various inocula. Each column represents the mean value of three replicates ± SE (vertical bars). Means with the same letters among different sources of inocula are not significantly different at the 0.05 level using the least significant difference (LSD) test

The fermentation profiles of various inoculum sources revealed significant differences in biofuel production after 48 h (Fig. 3A). Among the tested inocula, chicken manure significantly exhibited the highest production of both acetone and ethanol, reaching approximately 0.09 g/L and 0.08 g/L, respectively. Soil inoculum also showed a significant increase in acetone production (~0.09 g/L, while butanol production showed a significant increase in butanol production using cow dung inocula. Butyric and lactic acid concentrations were minimal across all inocula and were not significantly different from each other in most cases.

The sugar profile analysis across different inoculum sources revealed significant variations in initial sugar content, residual sugar, and glucose consumption (Fig. 3B). The initial sugar was exceeding 100 g/L. Residual sugar levels were lowest in banana peels and chicken manure inocula, indicating more efficient sugar utilization, while rumen and soil inocula showed higher residual sugar concentrations, suggesting incomplete fermentation. The consumed glucose was significantly increased using chicken manure, followed by banana peel and cow dung. These results suggest that chicken manure was more effective in glucose consumption, potentially enhancing fermentation efficiency.

### Determining the optimal concentration of banana peels for hydrogen production

**Fig. 4:**
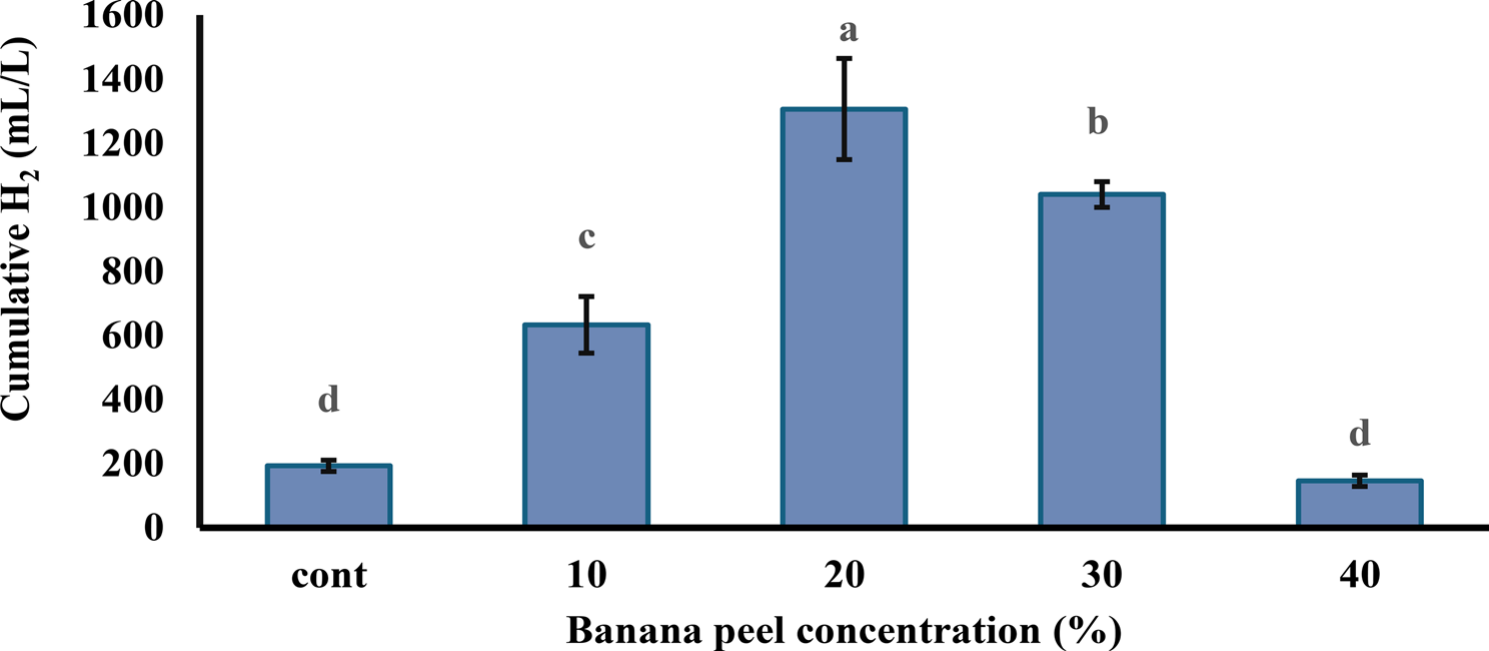
Impact of banana peels concentration inoculated with chicken manure on hydrogen production after 48 h. Each column represents the mean value of three replicates ± SE (vertical bars). Means with the same letters among different concentrations are not significantly different at the 0.05 level using the least significant difference (LSD) test

The results demonstrate that a banana peels concentration of 20% is optimal for maximizing hydrogen production. Conversely, higher concentrations of 40% were found to suppress hydrogen yield, suggesting an inhibitory effect at elevated levels. These observations are illustrated in Fig. 4.

**Fig. 5:**
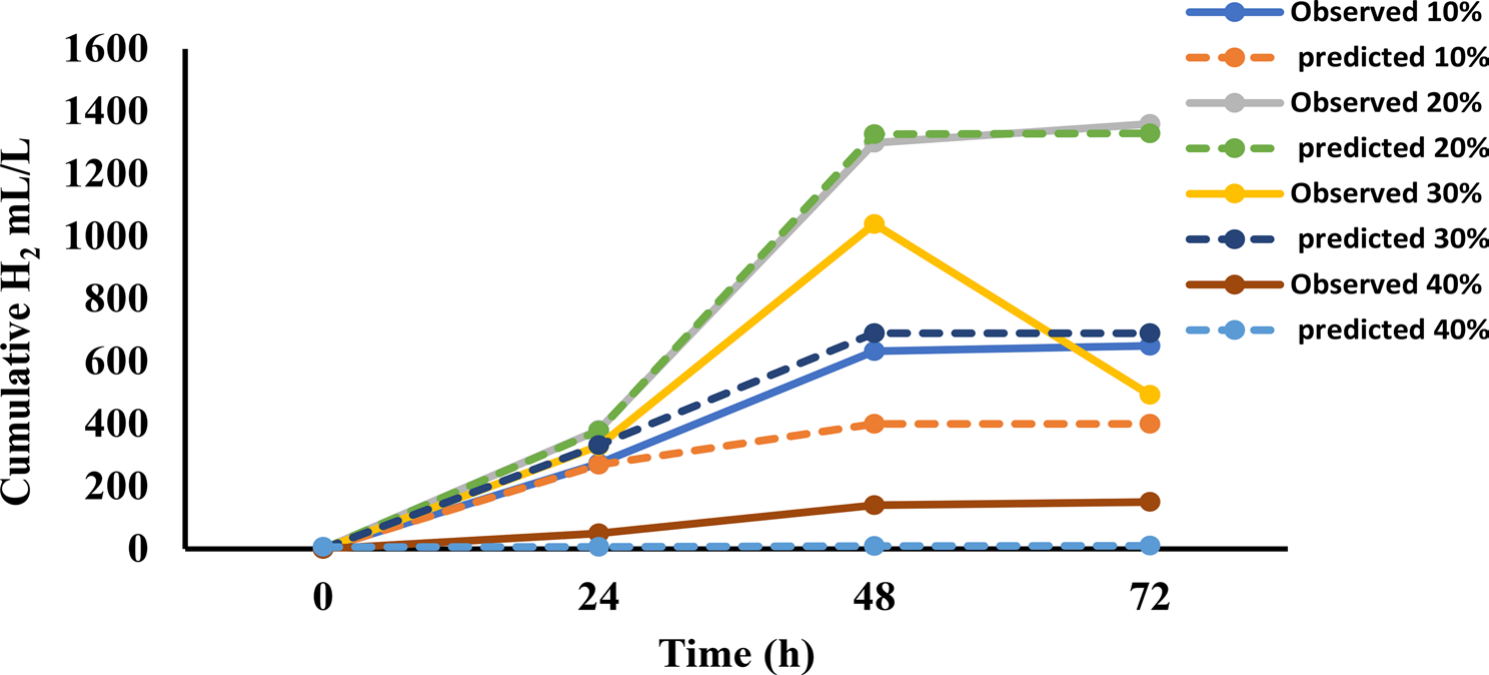
Cumulative hydrogen production (mL/L) over time (h) for different substrate concentrations. The graph compares observed and predicted values for 10%, 20%, 30%, and 40% concentrations. Solid lines represent observed data, while dashed lines indicate Gompertz model predictions

The Gompertz model was applied to predict cumulative hydrogen production across various banana peel concentrations, with results detailed in Fig. 5. The cumulative hydrogen production profiles over a 72-hour fermentation period revealed distinct trends across varying substrate concentrations (Fig. 5). Observed and predicted values were plotted for four substrate levels: 10%, 20%, 30%, and 40%. Hydrogen production increased steadily with time across all treatments, with the highest cumulative yield observed at the 20% concentration, reaching approximately 1400 mL/L. The 10% treatments showed moderate hydrogen production, while the 40% concentration resulted in comparatively lower yields, suggesting possible substrate inhibition at higher loading rates. Predicted values closely followed the observed trends, indicating a good model fit for all concentrations. The alignment between observed and predicted curves supports the reliability of the kinetic model used to simulate hydrogen production dynamics.

The kinetic modeling of hydrogen production from banana peels at varying substrate concentrations (10–40%) revealed notable differences in performance parameters (Table 2). At a 10% concentration, the model estimates a maximum cumulative hydrogen production (Hₘₐₓ) of 400 mL, a peak production rate (Rₘₐₓ) of 65.66 mL/h, and a lag phase of 19.69 h, achieving a strong fit (R² = 0.88). Increasing the concentration to 20% markedly boosts Hₘₐₓ to 1330 mL and Rₘₐₓ to 130 mL/h, with the lag phase extending slightly to 21.1 h; this scenario yields an improved model fit (R² = 0.99), indicating that a 20% concentration enhances hydrogen production efficiency. At 30%, however, Hₘₐₓ drops to 690 mL and Rₘₐₓ decreases to 110.6 mL/h, with a lag phase of 20.99 h and a solid fit (R² = 0.900), suggesting a decline in performance beyond 20%. At 40%, Hₘₐₓ reaches to 90 mL, but this value appears distorted by an outlier, accompanied by an extremely low Rₘₐₓ of 0.1 mL/h, a shortened lag phase of 3.34 h, and a poor model fit (R² = 0.25). These results, presented in Table 2, point to potential inhibitory effects at higher concentrations, undermining hydrogen production efficiency beyond the optimal 20% level.

**Table 2:** Kinetic parameters of hydrogen production from banana peels at different substrate concentrations

Banana peel concentration (%)	H _max_ (mL predicted)	*R*_max_ (mL/h ^− 1^ predicted)	λ (%) (h predicted)	*R* ^2^
10	400	65.66	19.69	0.88
20	1330	130	21.10	0.99
30	690	110.60	20.99	0.90
40	90	0.1	3.34	0.25

### Effect of different phenolic compounds on the growth of bacterial isolates

The bacterial isolates exhibited varied responses to five phenolic compounds: gallic acid, ferulic acid, pyrogallol, quercetin, and tannic acid, indicating diverse metabolic capabilities (Supplementary S1). Most isolates showed growth responses to gallic acid, ferulic acid, quercetin, and tannic acid, suggesting that these compounds are either metabolized or tolerated. Specifically, gallic acid supported growth in the majority of isolates, except for W2, W8, W9, W10, W16, W20, and W22, while quercetin was widely metabolized, with exceptions in W1 and W8. Ferulic acid also exhibited broad compatibility, with only W8 and W15 showing negative interactions. In contrast, pyrogallol elicited the weakest response, with only isolates W4, W6, W9, and W10 displaying a faint positive reaction (Supplementary S2). Tannic acid was generally well-tolerated, although W1 showed a weak positive response and W5 exhibited no growth (Table 3). These results highlight the diverse metabolic potential of the isolates, with pyrogallol presenting the most significant challenge, while gallic acid, ferulic acid, quercetin, and tannic acid were more readily utilized.

**Table 3:** Effect of different phenolic compounds on the growth of different bacterial isolates

Bacterial isolates	Gallic acid	Ferulic acid	Pyrogallol	Quercetin	Tannic acid
W1	+	+	-	-	+ weak
W2	-	+	-	+	+
W3	+	+	-	+	+
W4	+	+	+ weak	+	+
W5	+	+	-	+	-
W6	+	+	+	+	+
W7	+	+	-	+	+
W8	-	-	-	-	+
W9	-	+	+ weak	+	+
W10	-	+	+ weak	+	+
W11	+	+	-	+	+
W12	+	+	-	+	+
W13	+	+	-	+	+
W14	+	+	-	+	+
W15	+	-	-	+	+
W16	-	+	-	+	+
W17	+	+	-	+	+
W18	+	+	-	+	+
W19	+	+	-	+	+
W20	-	+	-	+	+
W21	+	+	-	+	+
W22	-	+	-	+	+ weak
W23	+	+	-	+	+
W24	+	+	-	+	+
W25	+	+	-	+	+
W26	+	+	-	+	+

‘+’ indicates positive growth, and ‘-’ indicates no growth

### Screening of bacterial isolates for cellulase and pectinase production

Twenty-six bacterial isolates were screened for cellulase and pectinase production. Among them, isolates W17, W18, W22, and W26 showed positive cellulase activity. Furthermore, isolates W13, W20, W23, W24, and W26 demonstrated pectinolytic activity on pectin agar medium. Notably, isolate W26 exhibited both cellulase and pectinase activities, indicating its versatility (Table 4).

**Table 4:** Screening of cellulase and pectinase enzymes production by different bacterial isolates

Bacterial isolates	Cellulase enzymes	Pectinase enzyme
W1	**-**	**-**
W2	**-**	**-**
W3	**-**	**-**
W4	**-**	**-**
W5	**-**	**-**
W6	**-**	**-**
W7	**-**	**-**
W8	**-**	**-**
W9	**-**	**-**
W10	**-**	**-**
W11	**-**	**-**
W12	**-**	**-**
W13	**-**	**+**
W14	**-**	**-**
W15	**-**	**-**
W16	**-**	**-**
W17	+	**-**
W18	+	**-**
W19	**-**	**-**
W20	**-**	**+**
W21	-	**-**
W22	+	**-**
W23	**-**	+
W24	**-**	+
W25	**-**	-
W26	**+**	**+**

‘+’ indicates positive activity, and ‘-’ indicates no activity

### Screening of bacterial isolates for H_2_ and biofuel production

Twenty-six bacterial isolates, sourced from cow rumen (R), soil (S), chicken manure (CM), cow dung (CD), and banana peels (BP), were individually evaluated for hydrogen (H₂) and biofuel production using banana peels as the substrate (Table 5). Among these, isolate W26, derived from cow rumen, exhibited the highest total hydrogen production at 1750 mL/L, with a production rate of 72.92 mL/h; however, it produced no detectable levels of acetone, butanol, or ethanol (ABE). Isolates W6 (1726.667 mL/L) and W5 (1060 mL/L) from chicken manure, along with W8 (1600 mL/L) from soil, also demonstrated substantial total hydrogen yields. These isolates achieved peak production rates of 71.92778 mL/h (W6), 44.16 mL/h (W5), and 66.67 mL/h (W8), reflecting their efficiency in rapidly generating hydrogen. In contrast, isolates W1, W4, W7, W9, and several others recorded no hydrogen production, marking them as the least effective. These results position W6, W5, and W8 as leading candidates for efficient hydrogen production.

For ABE production, isolate W17 from cow rumen stood out, yielding the highest total ABE concentration of 1.033 g/L, predominantly ethanol (0.930 g/L), indicating its versatility in both hydrogen and solvent production. The highest ABE yield were observed in W17 (1.033 g/L) from cow dung, W20 (1.151 g/L) from soil, and W22 (1.176 g/L) from cow rumen underscoring their capacity for rapid solvent synthesis. Isolate W8 also excelled in butyric acid production, reaching 0.167 g/L, while lactic acid production remained negligible across most isolates at 0.001 g/L. Overall, isolates W6, W5, and W8 emerged as the most promising for hydrogen production, while W17 and W20 showed significant potential for ABE output. These findings, detailed in Table 5, highlight the diverse capabilities of these isolates and their promise for sustainable biofuel production from banana peel waste.

**Table 5:** Screening of H₂ production (48 h) and acetone, butanol, ethanol, Butyric acid, and lactic acid production (72 h) by various bacterial isolates

Bacterial isolates	Isolation source	Total hydrogen, mL/L	Hydrogen rate, mL/h	Acetone, g/L	Butanol, g/L	Ethanol, g/L	Total ABE, g/L	Butyric acid, g/L	Lactic acid, g/L
W1	CM	0.000	0.000	0.055	0.010	0.054	0.119	0.011	0.001
W2	CM	140.0	5.830	0.090	0.000	0.109	0.199	0.005	0.001
W3	CM	193.3	8.050	0.054	0.000	0.013	0.067	0.010	0.001
W4	CM	0.000	0.000	0.113	0.000	0.043	0.156	0.008	0.001
W5	CM	1060	44.160	0.069	0.000	0.031	0.100	0.005	0.001
W6	CM	1727	71.920	0.103	0.007	0.042	0.152	0.019	0.001
W7	CM	0.000	0.000	0.062	0.000	0.043	0.105	0.006	0.001
W8	Soil	1600	66.670	0.093	0.000	0.008	0.101	0.167	0.001
W9	CM	103.0	4.200	0.020	0.000	0.010	0.100	0.000	0.000
W10	Soil	0.000	0.000	0.061	0.000	0.030	0.091	0.007	0.001
W11	Soil	1513	63.050	0.051	0.000	0.019	0.070	0.011	0.000
W12	Soil	0.000	0.000	0.115	0.000	0.007	0.122	0.005	0.001
W13	CD	860.0	35.830	0.080	0.000	0.033	0.113	0.012	0.001
W14	CD	0.000	0.000	0.069	0.000	0.054	0.123	0.016	0.001
W15	CD	1600	66.670	0.088	0.007	0.785	0.880	0.006	0.001
W16	CD	360.0	15.000	0.098	0.000	0.439	0.537	0.000	0.000
W17	CD	593.3	24.720	0.103	0.000	0.930	1.033	0.000	0.000
W18	CM	0.000	0.000	0.110	0.000	0.000	0.110	0.013	0.001
W19	CM	0.000	0.000	0.053	0.000	0.007	0.060	0.012	0.001
W20	Soil	0.000	0.000	0.097	0.000	1.054	1.151	0.006	0.001
W21	Soil	100.0	4.200	0.050	0.000	0.008	0.058	0.011	0.007
W22	CR	0.000	0.000	0.086	0.006	1.084	1.176	0.009	0.001
W23	BP	306.7	12.780	0.106	0.000	0.000	0.106	0.011	0.001
W24	BP	486.7	20.280	0.107	0.000	0.000	0.107	0.007	0.001
W25	BP	0.000	0.000	0.113	0.000	0.000	0.113	0.008	0.001
W26	CR	1750	72.920	0.000	0.000	0.000	0.000	0.007	0.001

### Phenotypic and genotypic characterization of hydrogen and biofuel producing bacteria

Twelve bacterial isolates, selected for their superior production of hydrogen, biofuels, cellulase, and pectinase, were characterized based on phenotypic traits. These isolates, originally obtained from cow rumen, soil, chicken manure, and banana peels, exhibited diverse morphological and biochemical profiles. Morphologically, the isolates included bacilli, streptobacilli, staphylococci, and short rods. On MacConkey agar, only isolate W21 displayed growth with pink colonies, indicative of lactose fermentation. Gram staining revealed that most isolates were Gram-positive, with W21 as the sole Gram-negative exception, further confirmed by a positive KOH test. Catalase activity was observed in isolates W8, W13, W20, W21, and W25, signifying the presence of the catalase enzyme. The majority of isolates tested positive for starch hydrolysis and gelatin hydrolysis, reflecting broad hydrolytic capabilities. Lipase activity was detected in W5, W8, W18, and W20, while urease production was evident in W5, W18, W24, and W25. None of the isolates produced hydrogen sulfide, and only W21 yielded a positive indole test result. Based on these phenotypic characteristics, W21 aligns with traits typical of the Enterobacteriaceae family, potentially resembling *Escherichia coli*. A comprehensive overview of the biochemical and physiological properties of these isolates is provided in Table 6.

**Table 6:** Biochemical test of some bacterial isolates able to grow on banana peels

Character	W5	W6	W8	W13	W17	W18	W20	W21	W22	W23	W24	W25
Shape	rods	rods	cocci	rods	cocci	cocci.	rods	rods	rods	rods	rods	rods
Growth on MacConkey agar	**-**	**-**	**-**	**-**	**-**	**-**	**-**	Pink color colonies	**-**	**-**	**-**	**-**
Gm stain	**+**	**+**	**+**	**+**	**+**	**+**	+	**-**	**+**	**+**	**+**	**+**
KOH	**-**	**-**	**-**	**-**	**-**	**-**	**-**	**+**	**-**	**-**	**-**	**-**
Catalase test	**-**	**-**	**+++**	**+**	**-**	**-**	**++**	**+++**	**-**	**-**	**-**	**+++**
Starch hydrolysis	**+**	**-**	**+**	**-**	**+**	**+**	**-**	**+**	**+**	**+**	**+**	**+**
Gelatin hydrolysis	**-**	**+**	**+**	**+**	**+**	**+**	**+**	**+**	**+**	**+**	**+**	**-**
Lipase test						**+**	**-**	**+**	**-**	**-**	**+**	**+**
Urease test	**+**	**-**	**-**	**-**	**-**	**+**	**-**	**-**	**-**	**-**	**+**	**+**
H_2_S production	**-**	**-**	**-**	**-**	**-**	**-**	**-**	**-**	**-**	**-**	**-**	**-**
Indole test	**-**	**-**	**-**	**-**	**-**	**-**	**-**	**+**	**-**	**-**	**-**	**-**
Carbohydrates test												
Glucose	**+++**	**+++**	**++**	**++**	**+++**	**+++**	**-**	**+++**	**++**	**-**	**+**	**++**
Sucrose	**+++**	**+**	**-**	**+++**	**+++**	**+++**	**-**	**+++**	**+**	**+++**	**+++**	**+**
Lactose	**-**	**-**	**-**	**-**	**-**	**-**	**-**	**+++**	**-**	**-**	**+**	**-**
Fructose	**+++**	**+++**	**-**	**++**	**+++**	**+++**	**-**	**+++**	**+**	**+++**	**+++**	**+**
Maltose	**+++**	**-**	**-**	**-**	**-**	**++**	**-**	**+**	**+**	**+**	**+**	**+**
Mannitol	**+++**	**+**	**+**	**++**	**++**	**+++**	**-**	**+++**	**-**	**+**	**+**	**+**
Glycerol	**+**	**-**	**-**	**+**	**-**	**+**	**-**	**+**	**-**	**+++**	**+++**	**+++**
Sorbitol	**+**	**-**	**+**	**-**	**-**	**+**	**-**	**+++**	**-**	**+**	**+**	**+**

‘+++’ indicates strong positive reaction, ‘++’ indicates medium positive reaction, ‘+’ indicates weak positive reaction, and ‘-’ indicates negative reaction

**Fig. 6:**
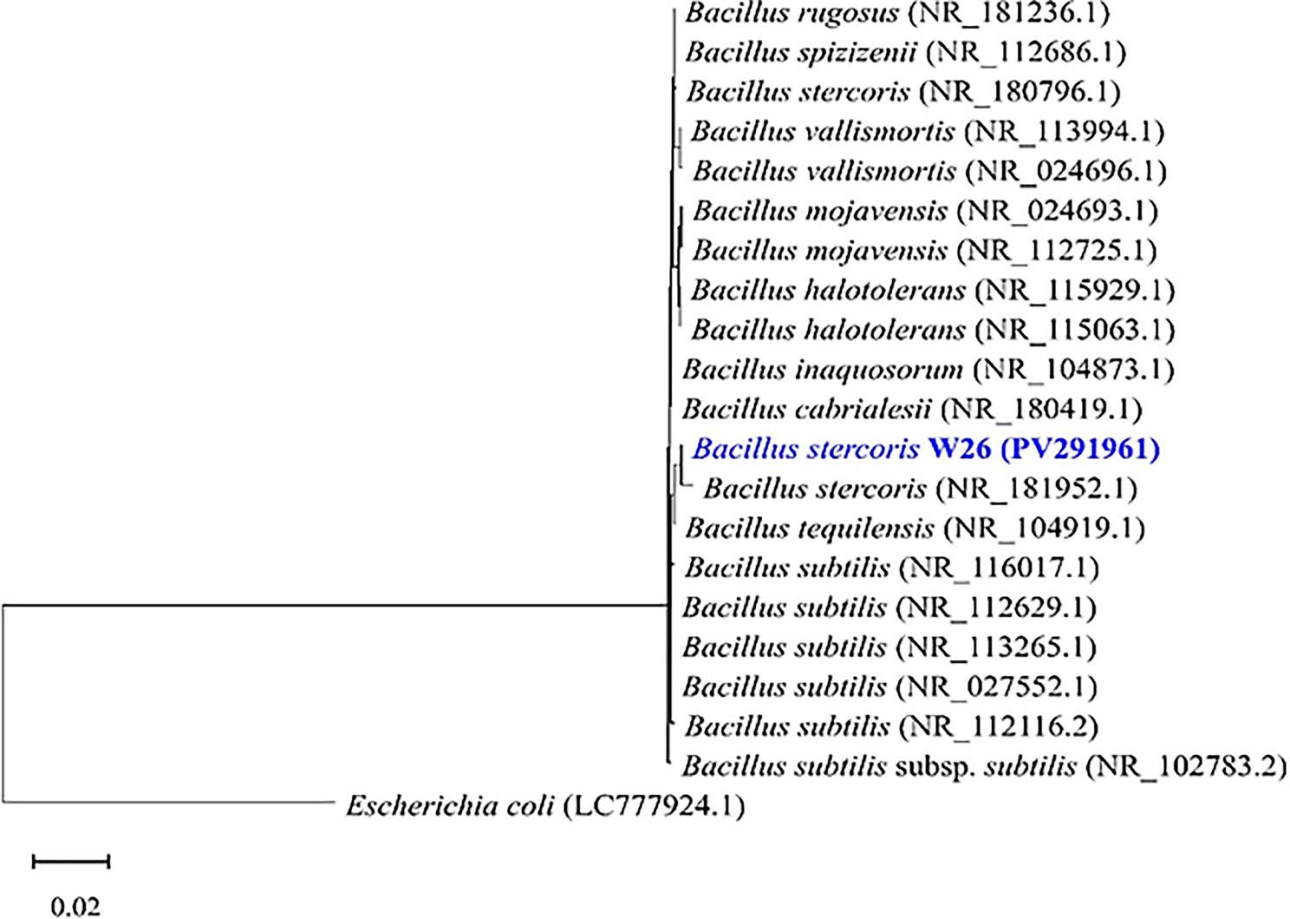
A phylogenetic tree based on neighbour-joining method using MEGA11. The scale bar represents 0.02 substitutions per nucleotide position. The isolate *Bacillus stercoris* W26 (PV291961) is highlighted in blue, showing its close relationship to other *Bacillus stercoris* strains. *Escherichia coli* (LC777924.1) were used as an out of group in the phylogenetic trees

Molecular identification by 16 S rRNA gene sequencing revealed that the almost complete 1419-bp 16 S rRNA gene sequence of W26 shares 99.93% identity with *Bacillus stercoris* (NR_181952.1). A phylogenetic tree of 16 S rRNA gene sequences was plotted (Fig. 6), and the annotated sequences were deposited in GenBank under the accession number PV291961.

## Discussion

The chemical composition of banana peels, rich in carbohydrates, proteins, and minerals including cellulose, hemicellulose, and lignin, explains their effectiveness as a robust carbon source for microbial fermentation, as the high COD and volatile solids provide ample organic matter for energy conversion, while the balanced C/N ratio of ~ 21.5 facilitates efficient microbial decomposition by preventing nitrogen limitation or carbon overload [35]. The slightly alkaline pH, though potentially insufficient for buffering acids during fermentation, likely contributes to initial microbial stability but highlights the need for pH monitoring to avoid process disruptions, warranting further investigation into pretreatment strategies. This composition makes banana peels a versatile substrate for microbial applications.

Banana peel extract’s viability as a cost-effective growth medium can be attributed to its inherent nutrient profile, which supports diverse bacterial growth similar to how agar derived from banana peels sustains pathogens like *Staphylococcus aureus*, *Escherichia coli*, and *Pseudomonas aeruginosa* [36]. This aligns with the observed microbial proliferation in our study, where the extract’s soluble organics likely mimicked natural nutrient-rich environments, enabling effective bioconversion. Beyond their role as a supporting microbial growth, banana peels are highly effective in bioconversion processes. These processes leverage the peels’ lignocellulosic structure to produce enzymes, organic acids, and other valuable compounds through solid-state or submerged fermentation, as seen in our enzymatic activity results, transforming agricultural waste into sustainable resources by enhancing substrate accessibility and microbial efficiency [37].

Given their bioconversion potential, banana peels are explored for bioenergy production, particularly hydrogen. The superior performance of chicken manure as an inoculum in hydrogen production can be explained by its diverse microbial consortium, including hydrogen-producing anaerobes, which likely synergized with the peels’ carbohydrates to achieve high yields, outperforming banana peels alone due to the latter’s limited indigenous microbes [38]. This microbial diversity in chicken manure probably facilitated better acidogenesis and acetogenesis pathways, leading to enhanced gas evolution compared to less diverse sources.

In comparison, cow dung produces only 283.3 mL/L and 8.0 × 10^4 CFU/mL, likely due to lower microbial diversity or fermentable material [38]. Such differences underscore how inoculum composition influences fermentation efficiency, as combining chicken manure with food waste enhances nutrient interactions and hydrogen yields by providing complementary substrates and microbes, mirroring our findings where chicken manure optimized sugar consumption and biofuel output [39]. Similarly, co-digestion of Napier grass and cow dung with *Clostridium butyricum* provides alternative hydrogen production pathways by introducing specialized fermenters that overcome substrate limitations [40]. However, soil’s microbial diversity proved insufficient for effective hydrogen production due to suboptimal anaerobic conditions and lack of specialized hydrogen producers, resulting in no detectable gas despite activity; this contrasts with banana peels and rumen fluid, which offered moderate yields likely because of their inherent or introduced anaerobes, improvable through pretreatment to reduce inhibitors or enhance microbial adaptation. Rumen inoculum outperforms sludge in fermentation [41], and acid-treated rumen fluid enhances hydrogen yields from paper waste in batch reactors [42]. These comparisons suggest that the moderate yields from rumen in our study stemmed from its natural enrichment in fiber-degrading microbes, though less effective than chicken manure without pretreatment. Among these inocula, chicken manure remains the most effective, underscoring the importance of inoculum selection. To optimize hydrogen production, the concentration of banana peels as a substrate is critical.

The identification of 20% as the optimal banana peels concentration for hydrogen production reflects a balance where sufficient substrate supports microbial growth without overwhelming the system, as evidenced by the Gompertz model’s high fit (R² = 0.99), with Hmax and Rmax peaking due to efficient carbon utilization; this surpasses lower concentrations (e.g., 10%) by providing more fermentable sugars without inhibition, while higher levels (30–40%) likely induced toxicity from accumulated acids or phenolics, reducing yields [43, 44]. This substrate-dependent pattern aligns with general fermentation principles, where higher concentrations enhance carbon utilization by hydrogen-producing bacteria, boosting yields, but excessive levels may reduce output due to inhibitory compounds accumulating and disrupting microbial metabolism [45]. Further research into these compounds’ effects is needed to optimize substrate concentrations. These results with other fruit peels, such as watermelon and melon peels achieving high biohydrogen yields with *C. butyricum* (H_max_ 991.00–1062.67 mL/L) [46, 47], suggest broader applications for banana peels in similar hydrogen production systems.

The presence of inhibitory compounds, particularly phenolics, in banana peels influences their bioenergy potential. Banana peels are rich in phenolic compounds (227–689 mg gallic acid equivalent/100 g dry weight), ranking second among fruit peels [48, 49]. Key compounds like ferulic acid and flavan-3-ols, extracted via ultrasound at 60 °C for 30 min (18.21–35.06 mg/g), exhibit antimicrobial properties effective against Gram-positive and Gram-negative bacteria, making them promising natural antimicrobial agents for pharmaceutical applications [50–55]. Tests with 26 isolates showed that gallic acid, ferulic acid, quercetin, and tannic acid were effective, while pyrogallol performed poorly, and tannic acid’s efficacy varied (Table 3). Further studies on these compounds’ mechanisms could clarify their antimicrobial potential.

In addition to phenolic tolerance, bacterial isolates from banana peels demonstrate enzymatic capabilities for biofuel production. The cellulase production by isolates W17, W18, W22, and W26 likely arises from their adaptation to lignocellulosic substrates, enabling efficient conversion of cellulose into fermentable sugars for bioethanol, as the clear hydrolysis zones indicate active enzyme secretion; this mirrors high-yield conversions achieved with sugarcane bagasse using aqueous ammonia soaking and laccase-mediator hydrolysis, where similar pretreatments enhance accessibility [56], and with lignocellulosic waste using cockroach-gut bacteria and yeast fermentation, suggesting shared evolutionary adaptations in gut-derived microbes [57]. This enzymatic versatility extends to pectinase production, with isolates W13, W20, W23, W24, and W26 efficiently degrading pectin, increasing fermentable sugar yields and enhancing bioethanol production. Combining pectinase and xylanase further improves bioethanol production from horsetail waste with *Saccharomyces cerevisiae* [57]. The dual activity in W26 can be attributed to its rumen origin, where such enzymes are naturally selected for fiber breakdown, offering a safer, cost-effective, and eco-friendly alternative to chemical methods for waste management and bioethanol production through microbial fermentation of banana peels and other fruit waste [58–61]. Similarly, *Bacillus subtilis* strain NG 105 (MN493055) efficiently produces these enzymes using mosambi peel, suggesting banana peels could support comparable enzyme yields due to analogous pectin and cellulose content [62].

The exceptional performance of isolate W26 (*Bacillus stercoris*, 99.93% 16 S rRNA match) in hydrogen production reflects its metabolic robustness, likely derived from rumen adaptations that favor anaerobic fermentation pathways, enabling high yields without ABE byproducts; this contrasts with isolates like W6, W5, and W8, whose strong hydrogen outputs may stem from similar but less optimized pathways. Bacterial isolates W6, W5, and W8 also show strong hydrogen yields, while W17 (from cow dung) and W20 lead in acetone-butanol-ethanol (ABE) production, with W17 excelling in ethanol and W8 in butyric acid. Further research into microbial interactions could enhance hydrogen production efficiency. Fruit wastes, including banana peels, support the production of reducing sugars, bioethanol, and hydrogen [63–67]. For instance, marine *Citrobacter* sp. E4 produces ethanol from fruit waste [64], while *Bacillus coagulans* MO11 yields 1.634 mol H₂/mol hexose from molasses and wastewater [66]. Isolate W21 (*E. coli*)’s ability to metabolize gallic acid, quercetin, and tannic acid but not pyrogallol suggests selective enzymatic degradation, possibly conflicting with tannic acid’s reported inhibition of *E. coli* biofilm formation due to concentration-dependent effects or strain-specific tolerances [68]. These findings, combined with the capabilities of isolates W26, W17, and W20, highlight the potential of microbial metabolism for sustainable biofuel production. Alternative hydrogen production pathways, like bacterial co-digestion, show promise but need further validation to optimize yields.

## Conclusions

This study highlights the potential of banana peels as a sustainable substrate for biohydrogen and biofuel production through microbial fermentation. Chemical analysis revealed high levels of fermentable sugars and volatile solids, indicating their suitability for anaerobic digestion. Among different inocula tested, chicken manure significantly boosted hydrogen yield, with bacterial isolates W6, W5, and W8 emerging as top hydrogen producers. Bacterial isolate W17 demonstrated notable ABE productivity, while bacterial isolate W26, identified as *Bacillus stercoris*, exhibited cellulase and pectinase activity. Optimal hydrogen production was achieved with a 20% banana peels loading, as confirmed by kinetic modeling using the Gompertz model. The study also highlighted the metabolic versatility of bacterial isolates in tolerating phenolic compounds and producing biofuels. These results emphasize the potential of agricultural waste in renewable energy applications and support the advancement of integrated bioprocesses for waste valorization. Future research should focus on multi-variable optimization, scale-up possibilities, and co-culture strategies to further enhance biohydrogen yields and process efficiency.

## Supplementary Information

Below is the link to the electronic supplementary material.

Supplementary Material 1

## Data Availability

16 S rRNA nucleotide sequence of the bacterial isolate **Bacillus stercoris** strain w26 isolated from Cow Rumen was deposited in the GenBank database and assigned the accession number PV291961 [https://www.ncbi.nlm.nih.gov/nuccore/2930187707](https:/www.ncbi.nlm.nih.gov/nuccore/2930187707) . All the analyzed and generated data are included in this study.

## Abbreviations

ABE: Acetone-Butanol-Ethanol
H_max_: Maximum Hydrogen Production
R_max_: Maximum Production Rate
16S rRNA: 16 S Ribosomal RNA
FAO: Food and Agriculture Organization
TS: Total Solids
VS: Volatile Solids
COD: Chemical Oxygen Demand
APHA: American Public Health Association
BPEA: Banana Peel Extract Agar
CMC: Carboxymethyl Cellulose
DNA: Deoxyribonucleic Acid
CFU: Colony-Forming Units
BP: Banana Peels
S: Soil
CR: Cow Rumen
CM: Chicken Manure
CD: Cow Dung
R²: Coefficient of Determination
gVS: Grams of Volatile Solids
PBEA: Peel Extract Agar
C/N: Carbon-to-Nitrogen Ratio
λ(Lambda): Lag Time
