# Supplementary material for: Sustainable biohydrogen production from banana peels using microbial fermentation
Source: BMC Biotechnol. 2025 Dec 13;26:5. doi: 10.1186/s12896-025-01080-3 (PMC12809973; doi:10.1186/s12896-025-01080-3)

**Harnessing banana peels using microbial fermentation for sustainable and renewable biofuels production**

**Shymaa Ryhan Bashandy*****^1^, Wafaa Abdelnaser Sleem^1^, Mohamed Hemida Abd-Alla**^1^ and David Mamdouh Khalaf^1^**

***Corresponding author:**

**** http://orcid.org/0000-0003-0415-9409**

**Running title: biofuel production from banana peels**

**S1. Effect of phenolic compounds (gallic acid (A), ferulic acid (B), quercetin (C), and tannic acid (D)) of the growth of 26 bacterial isolates**


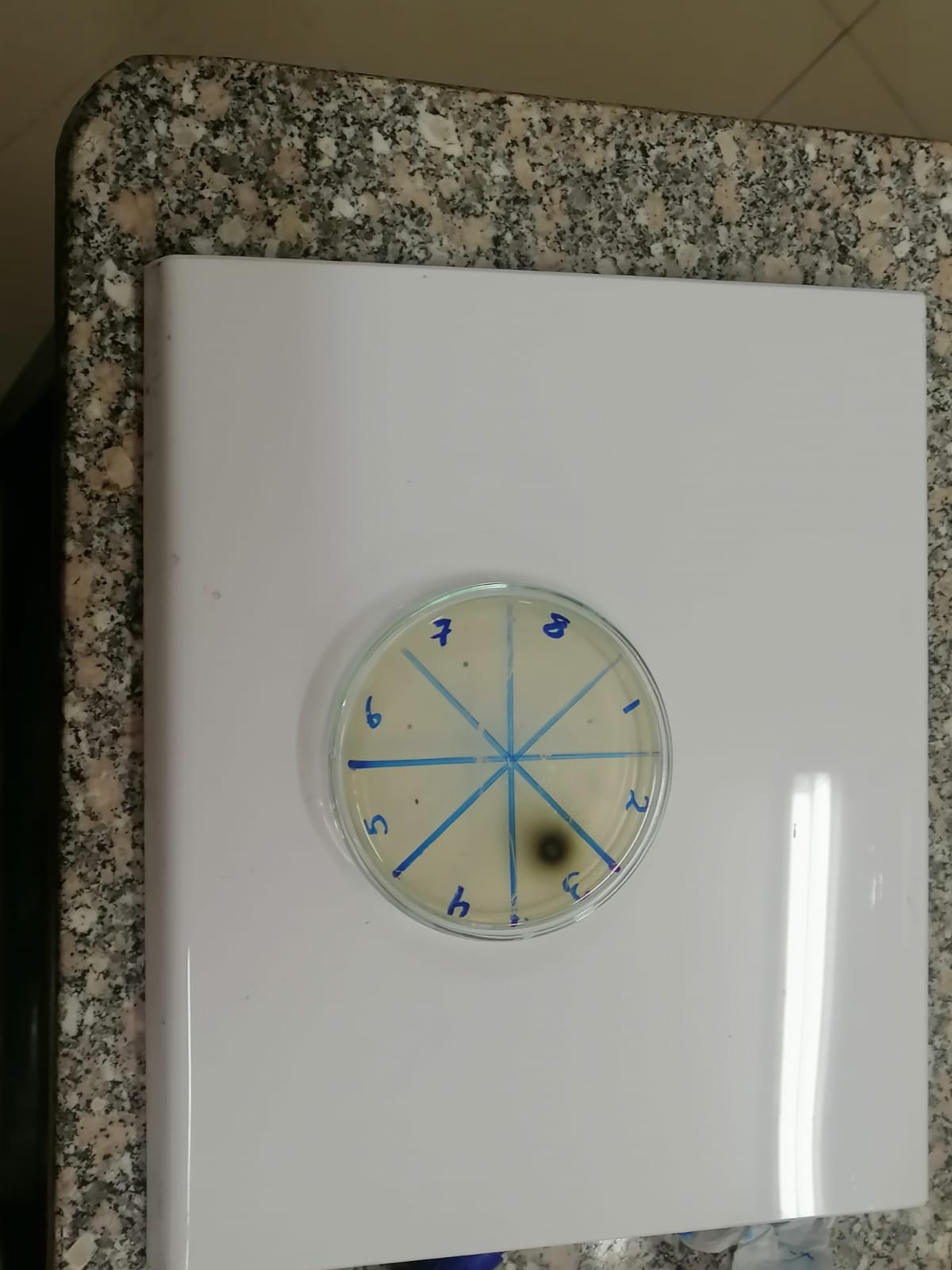

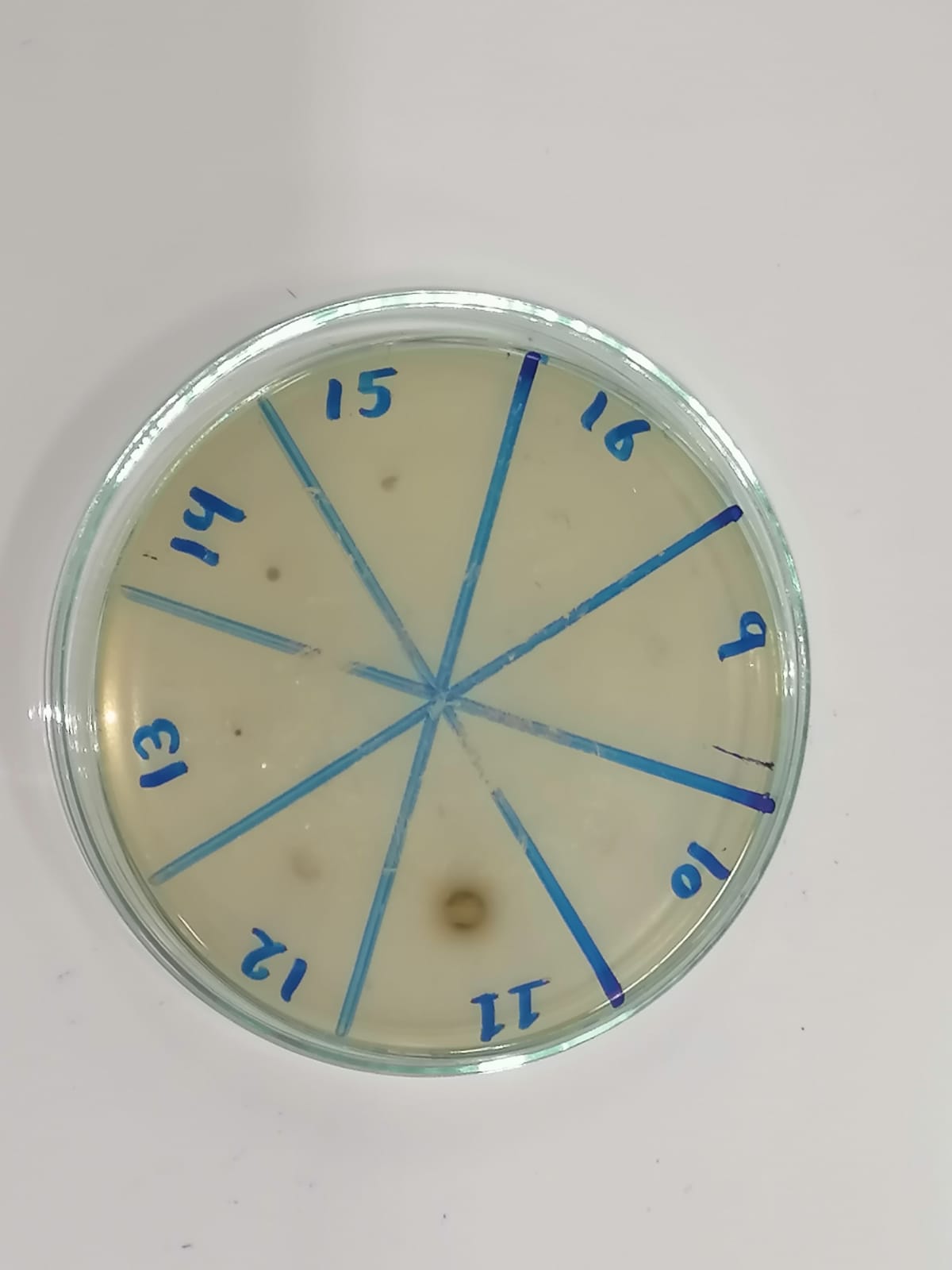

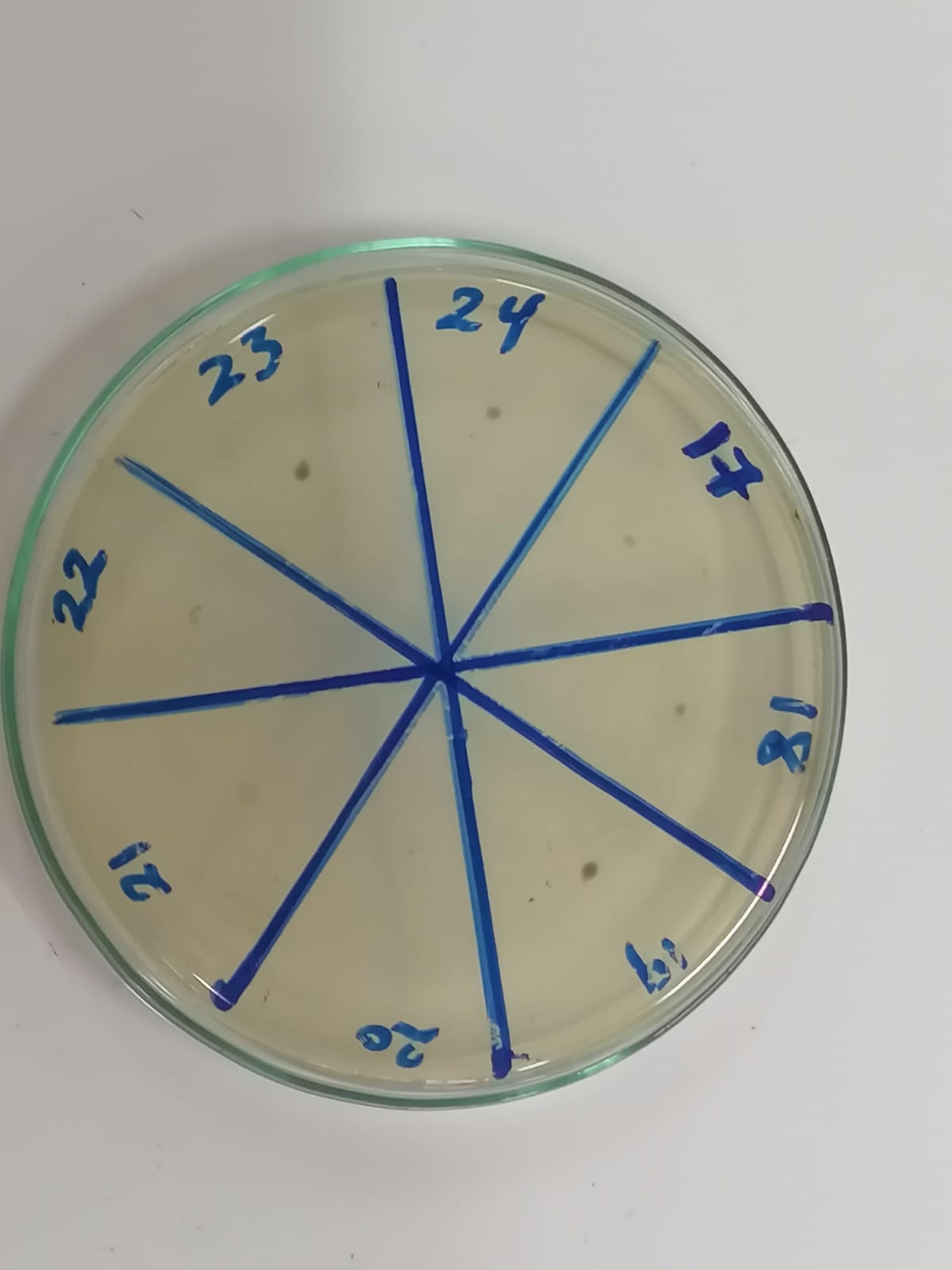

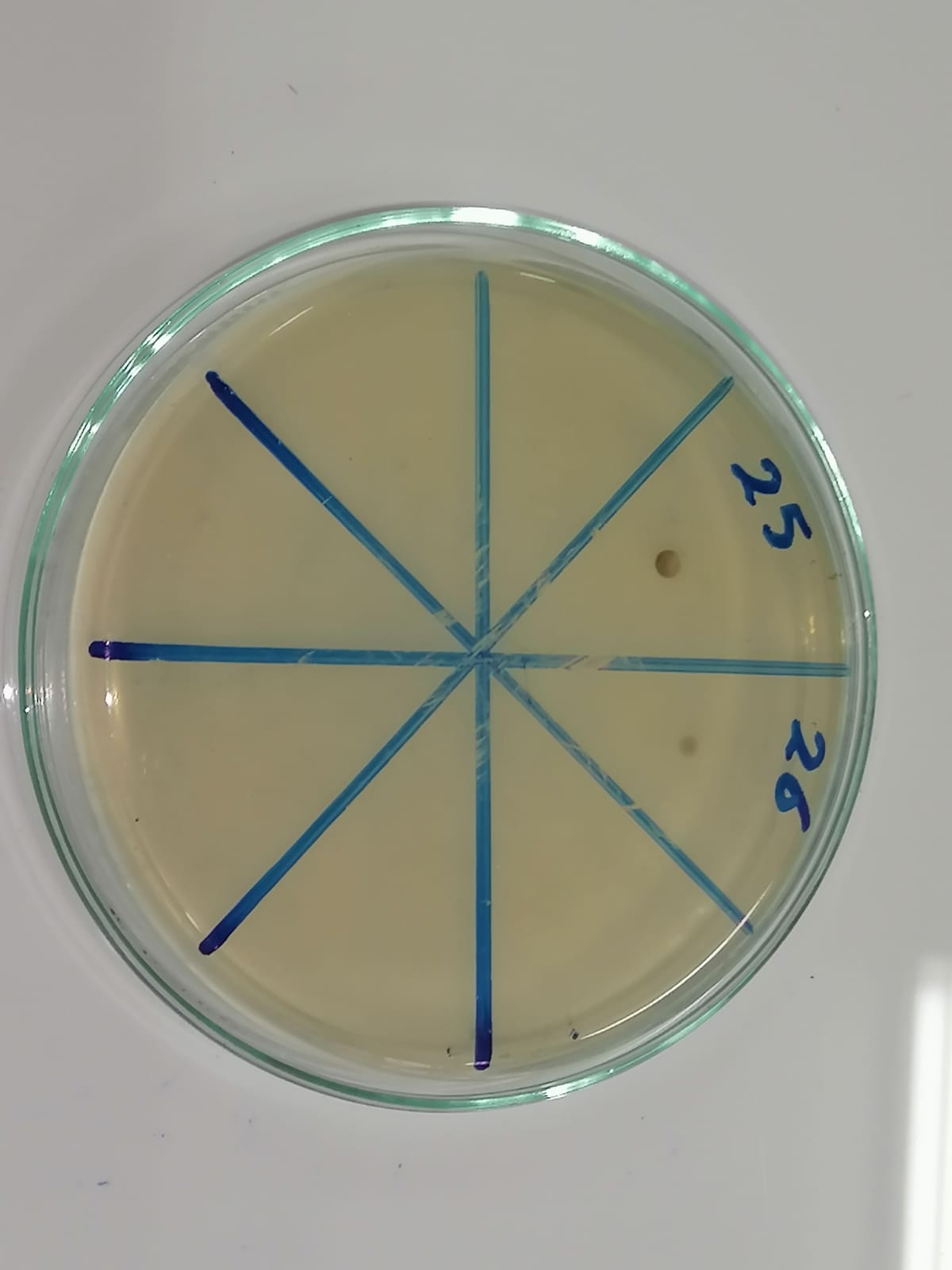


**S1. A**


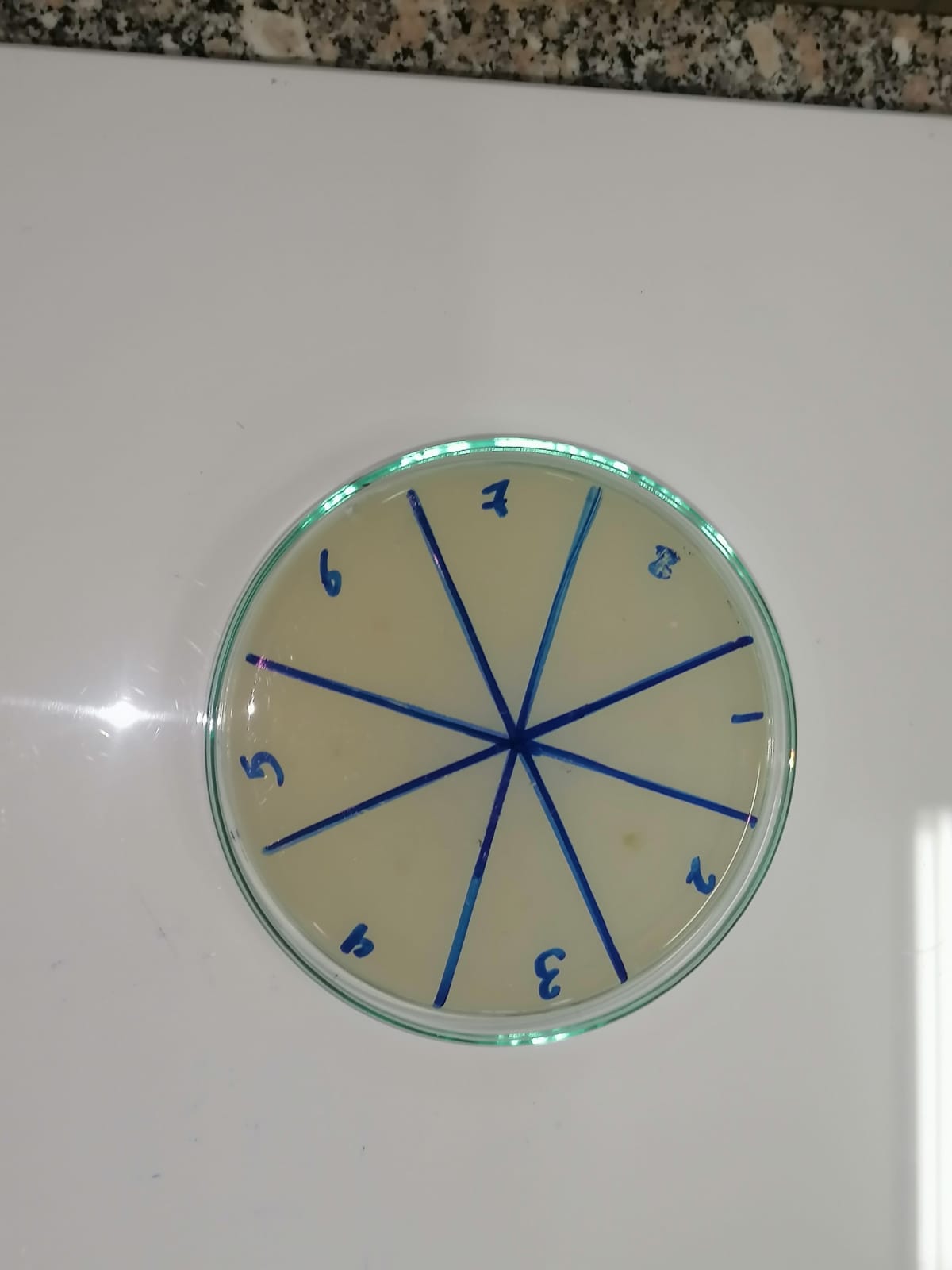

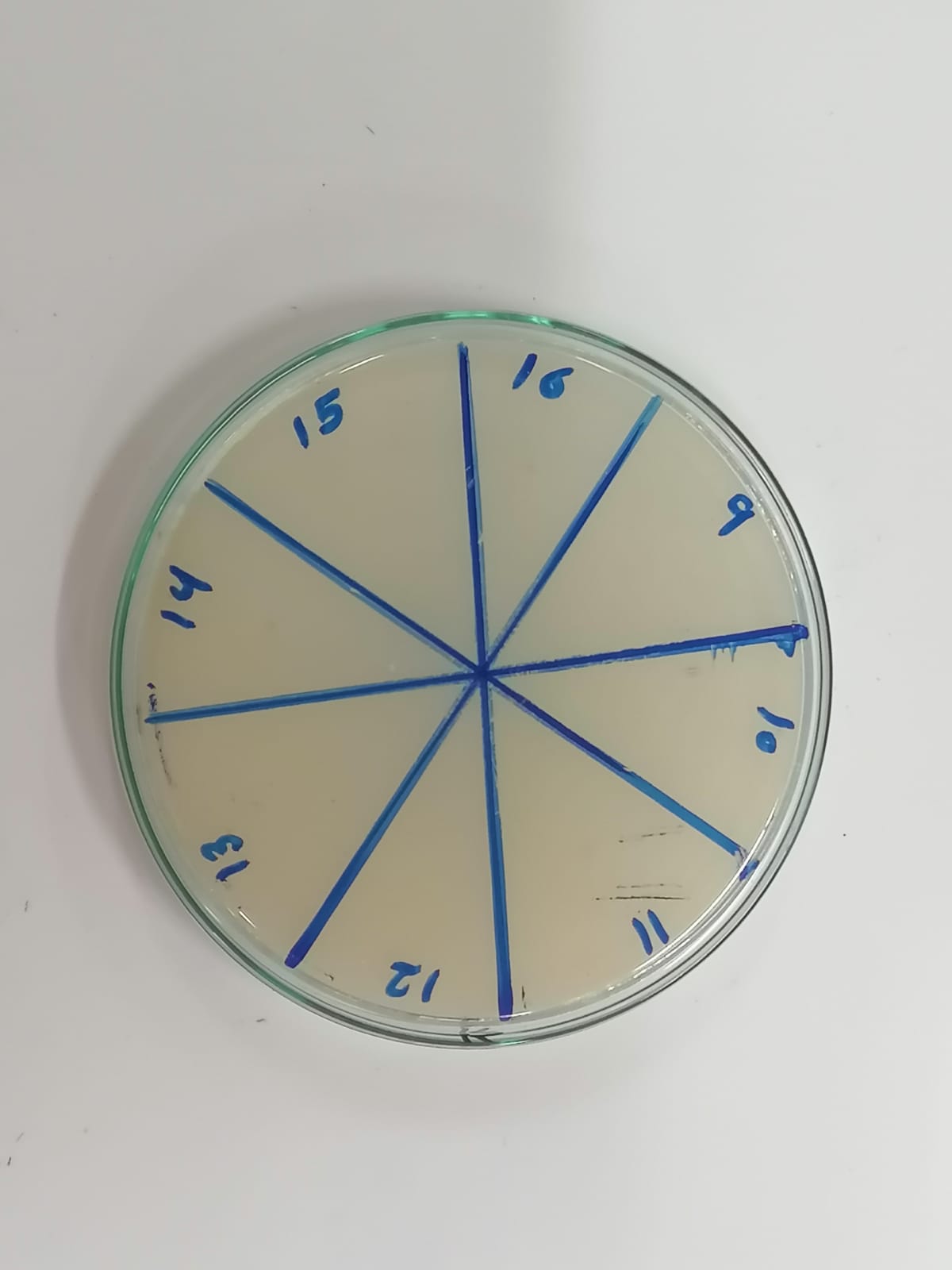

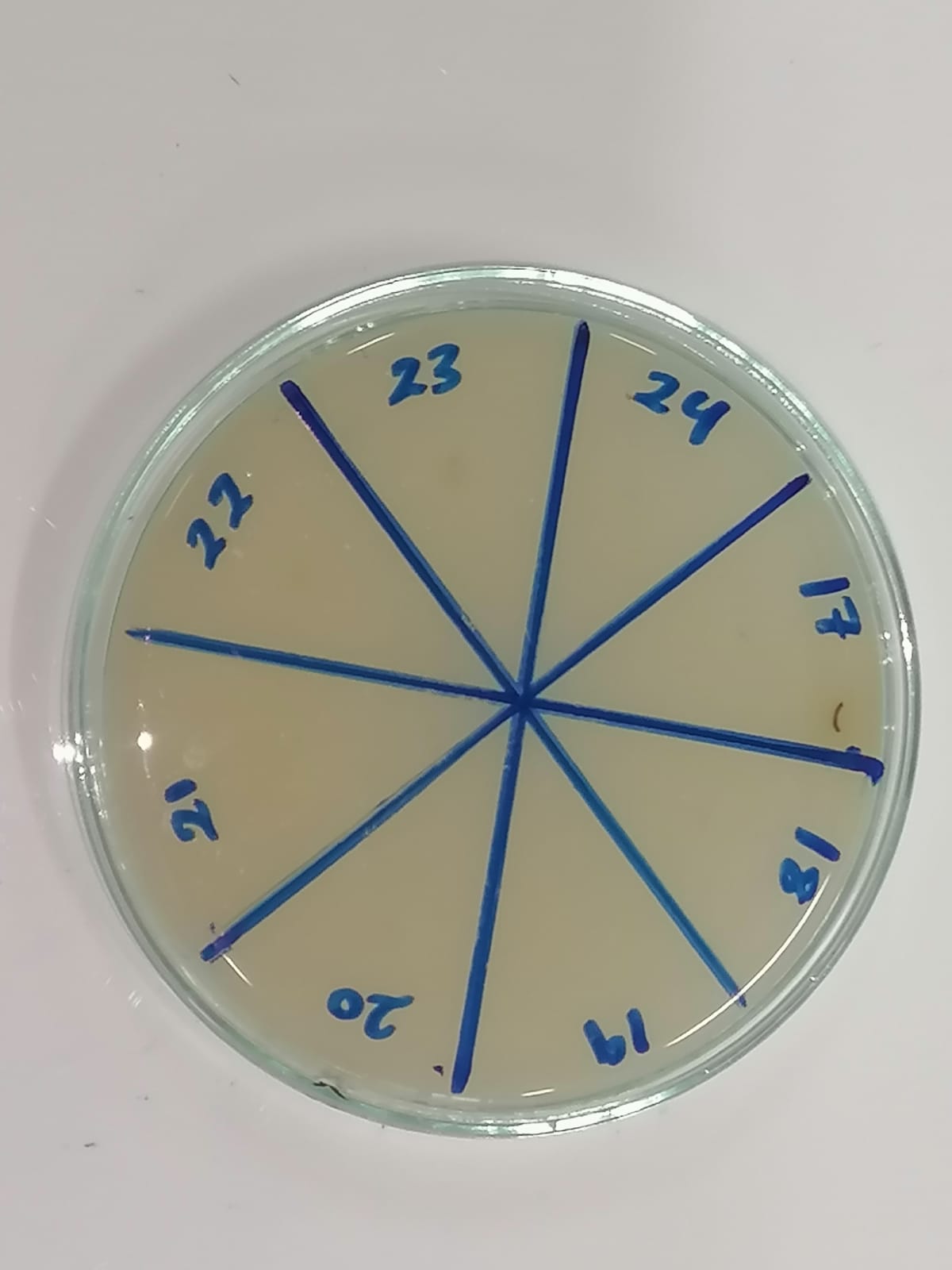

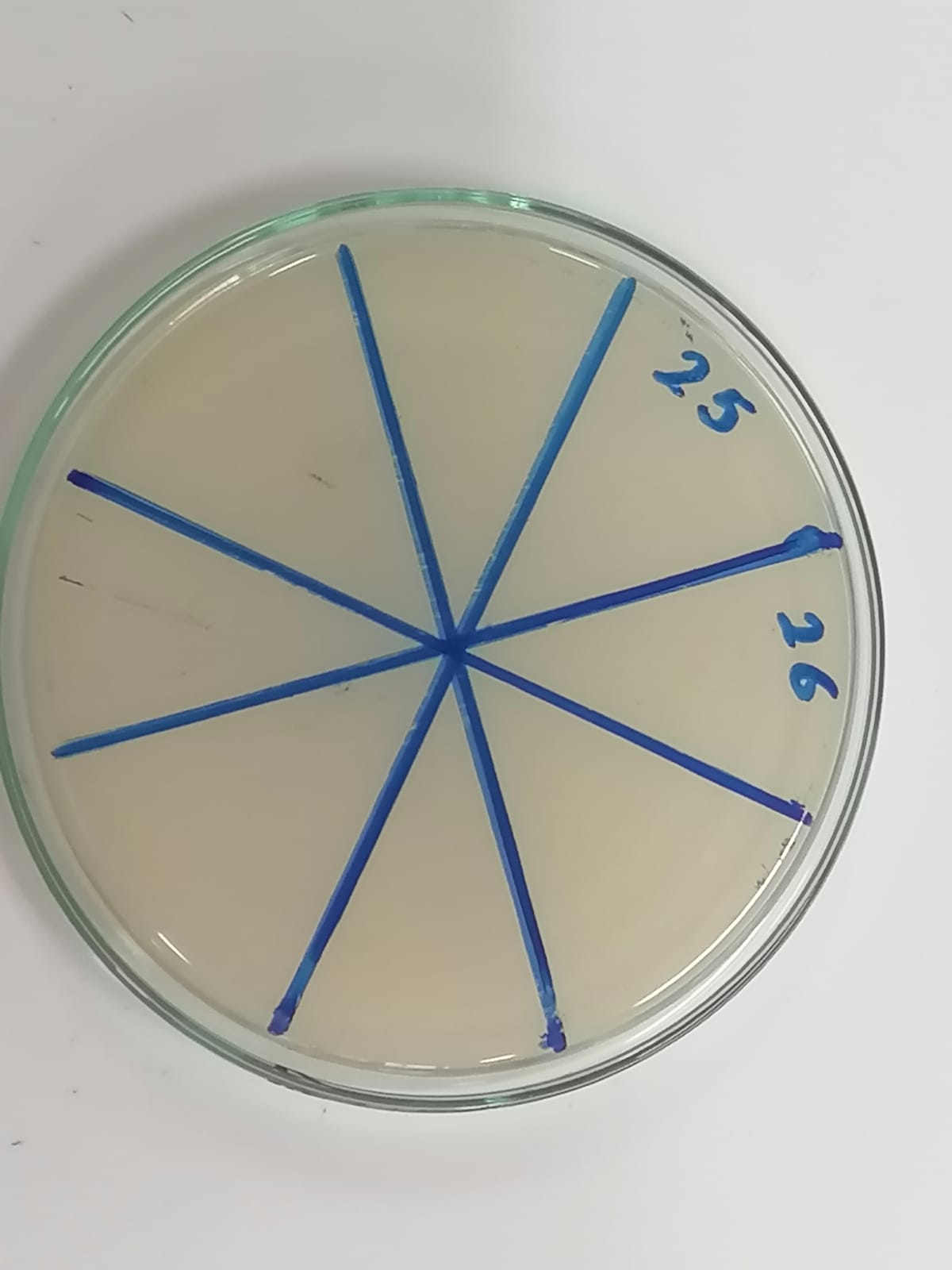


**S1. B**

**Quercetin**


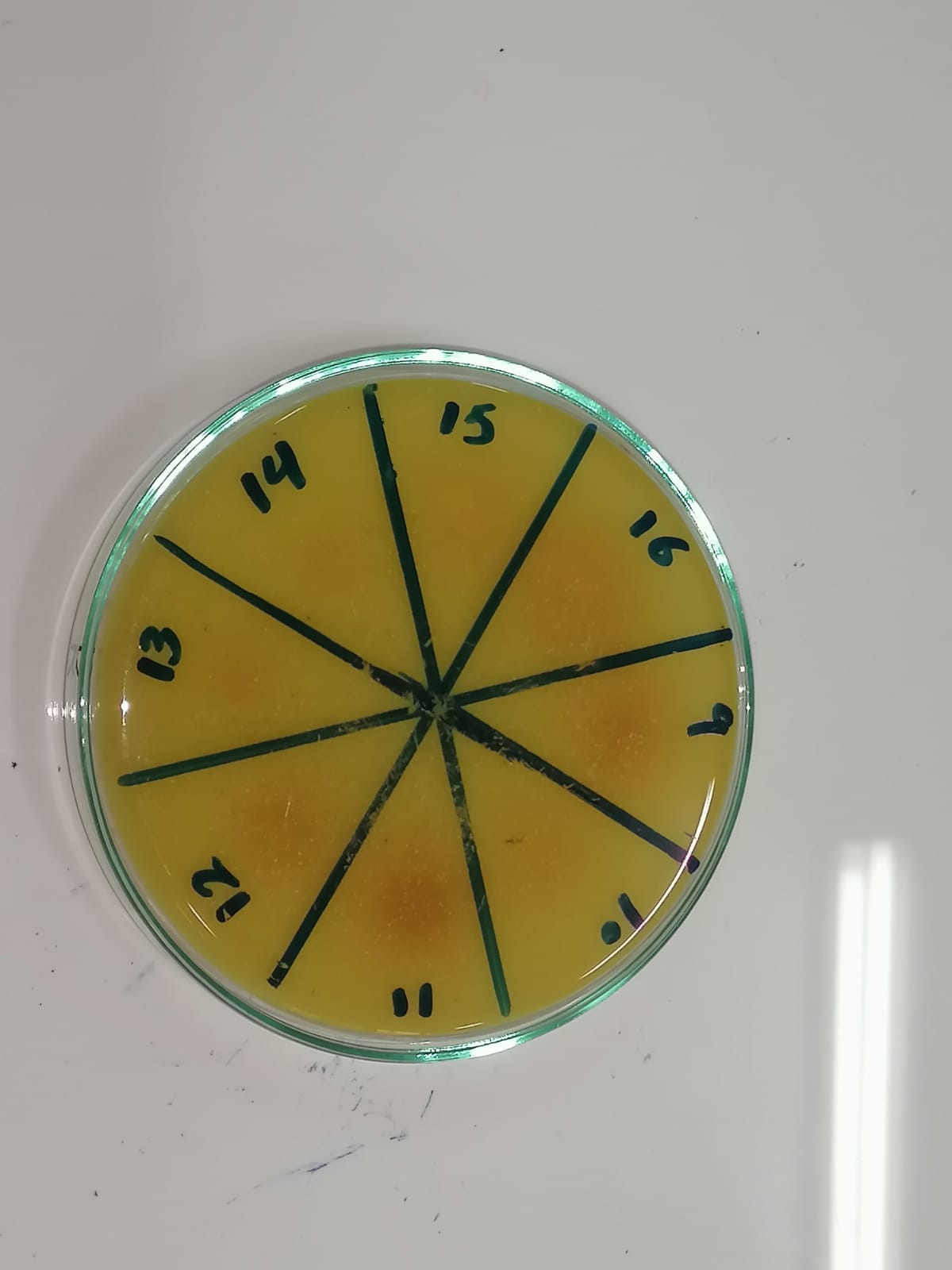

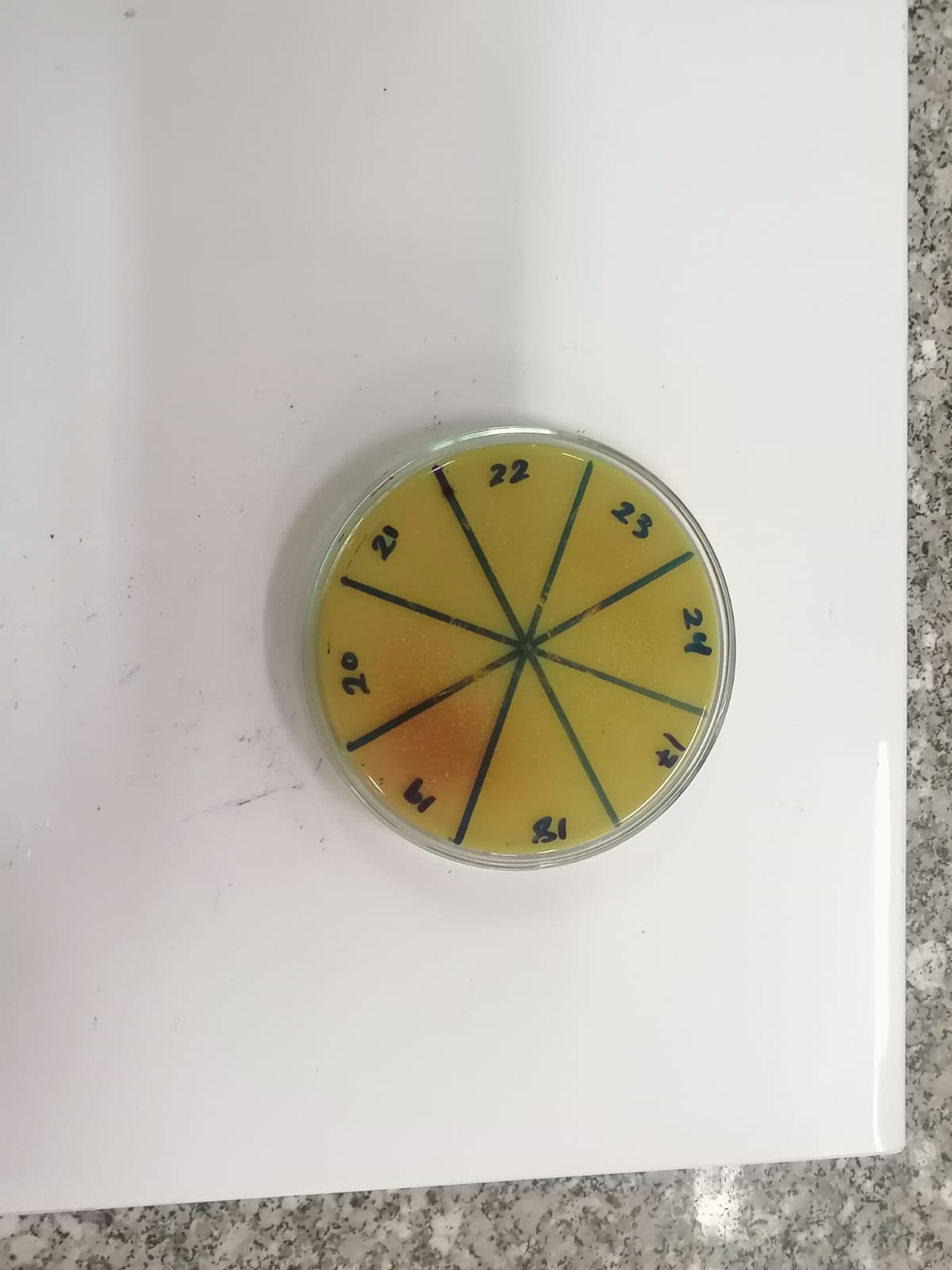

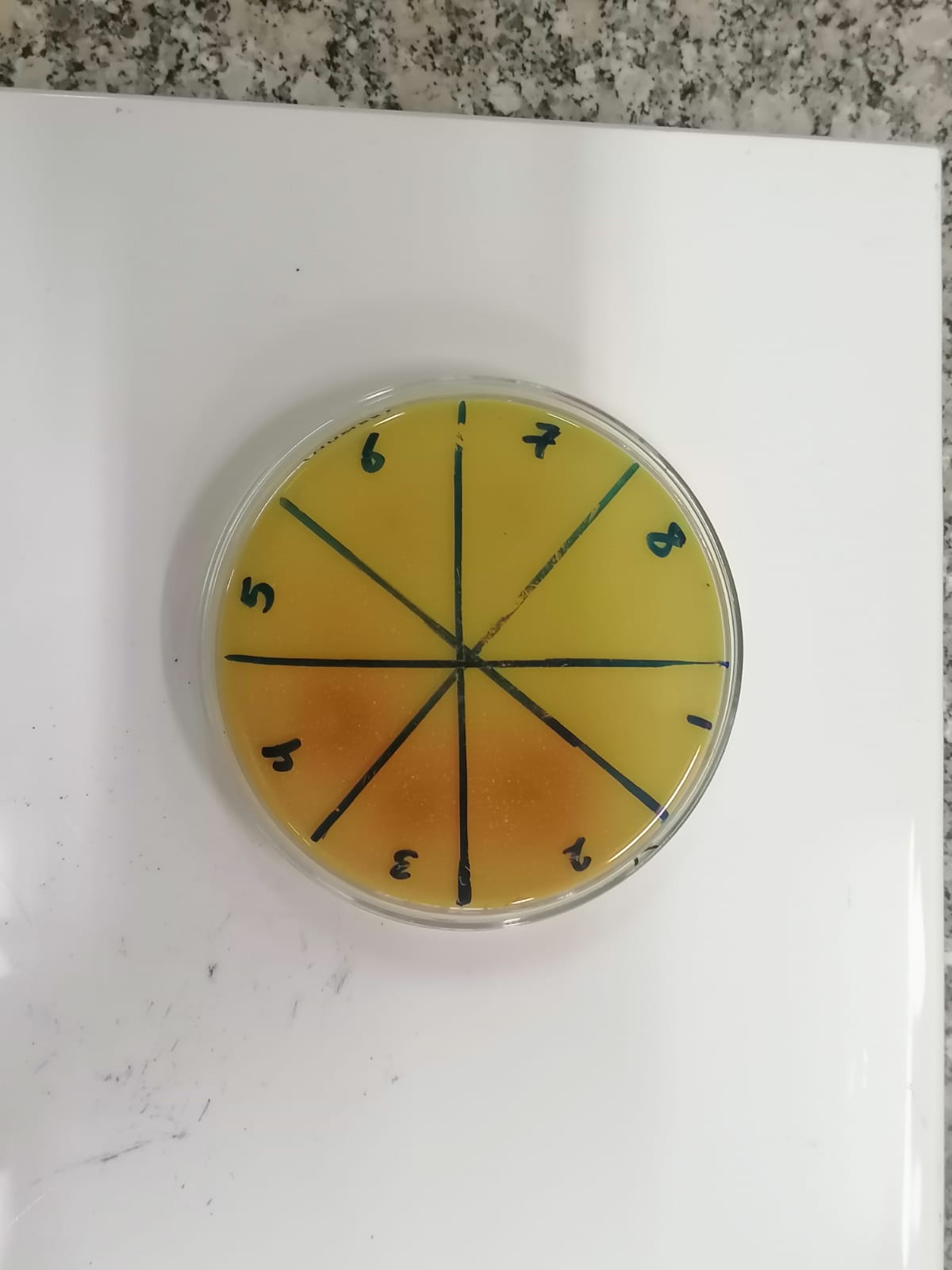

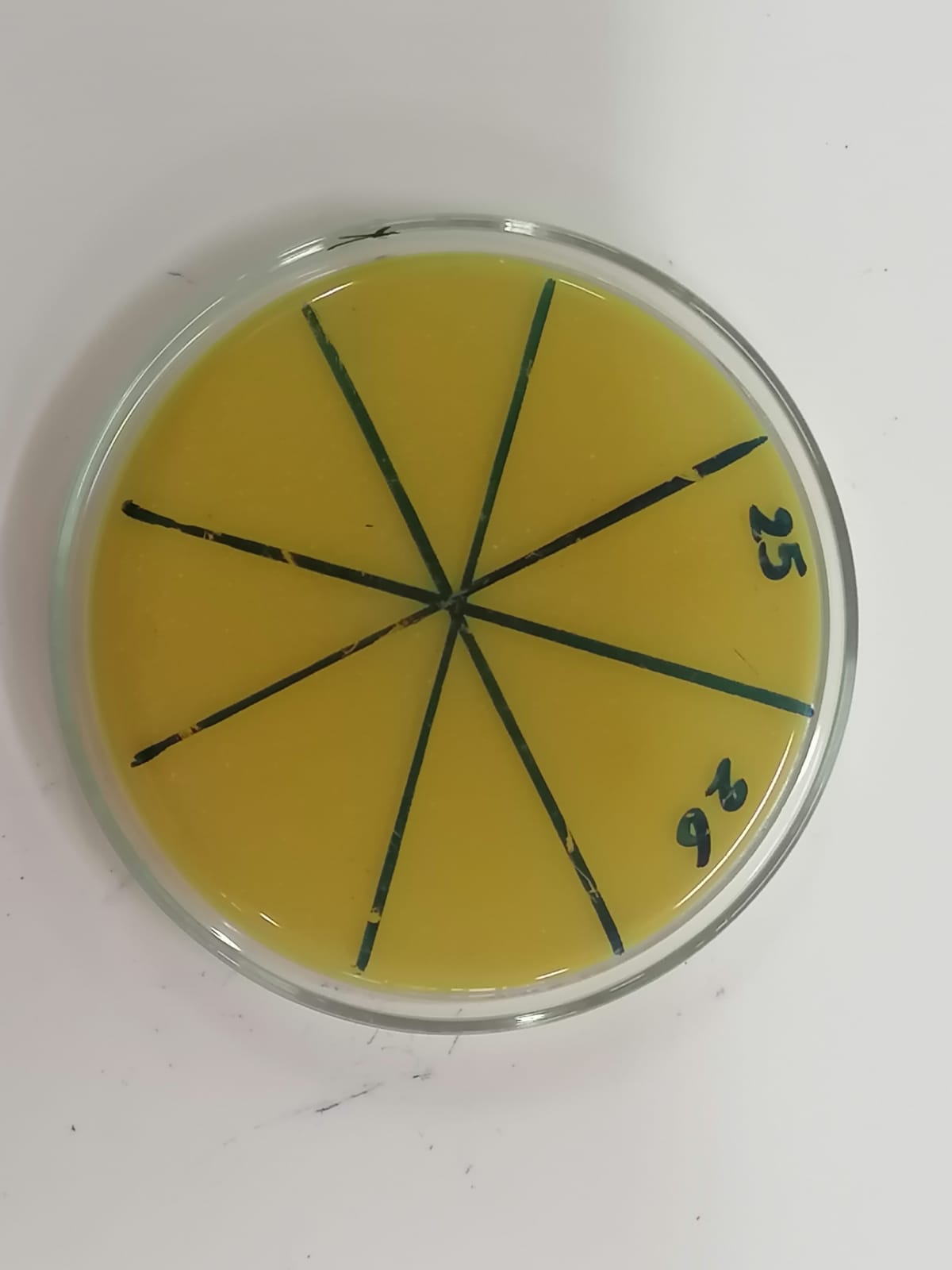


**S1.C**

**Tannin (Tannic acid)**


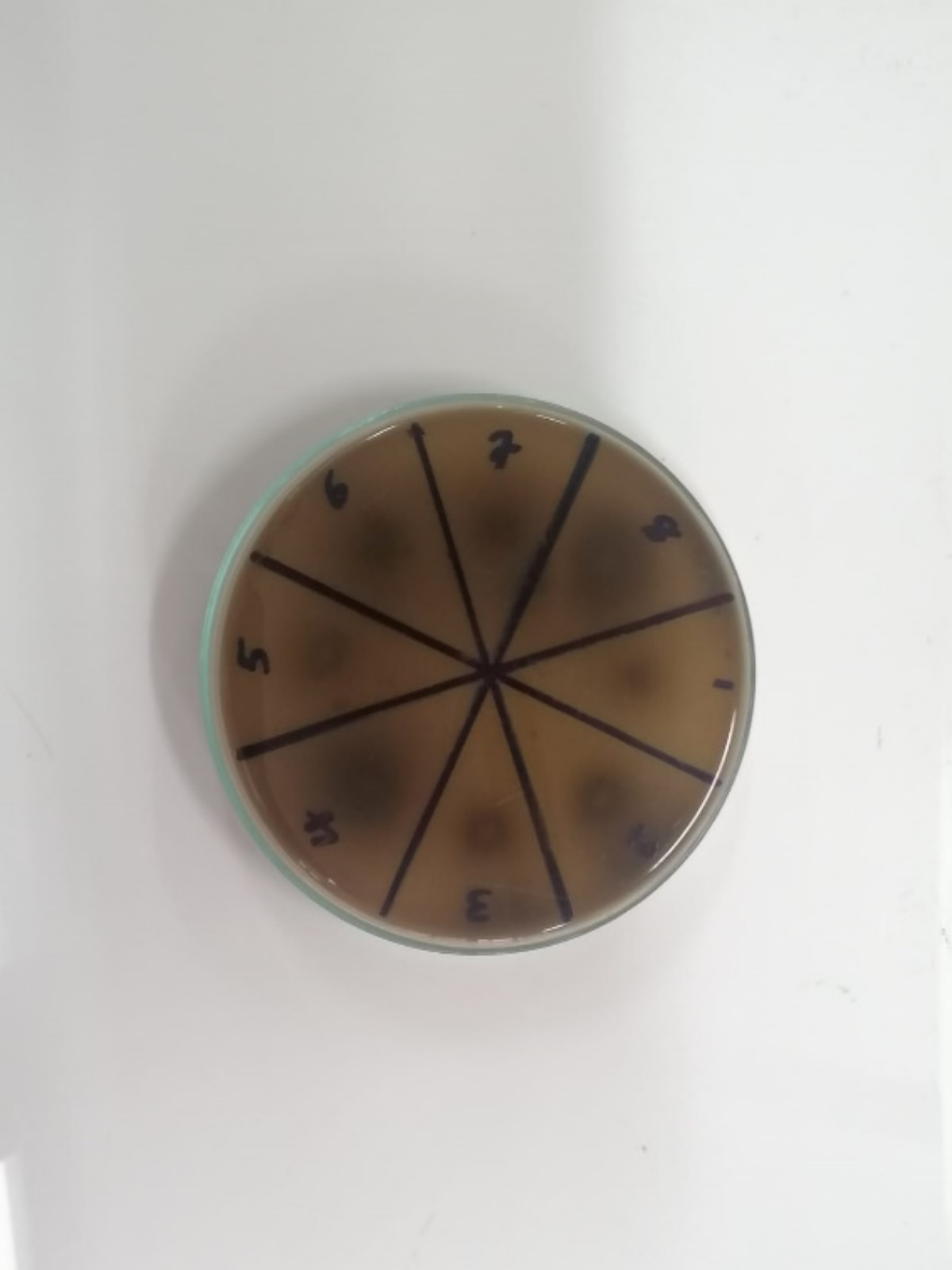

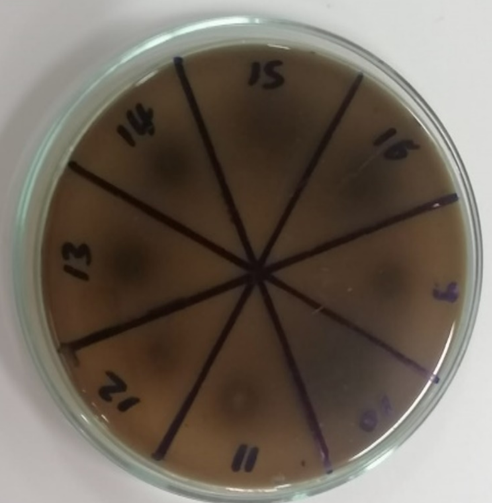

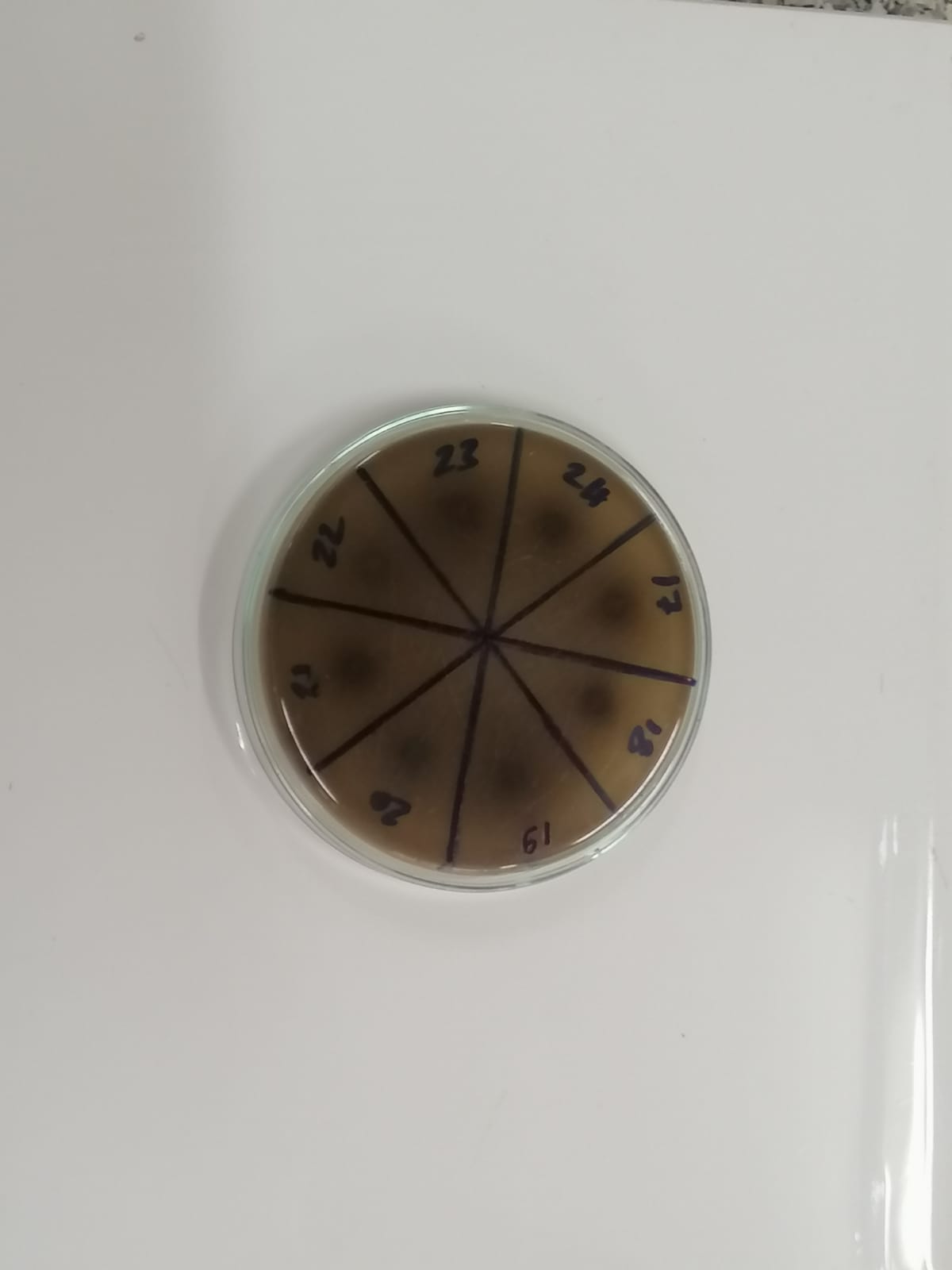

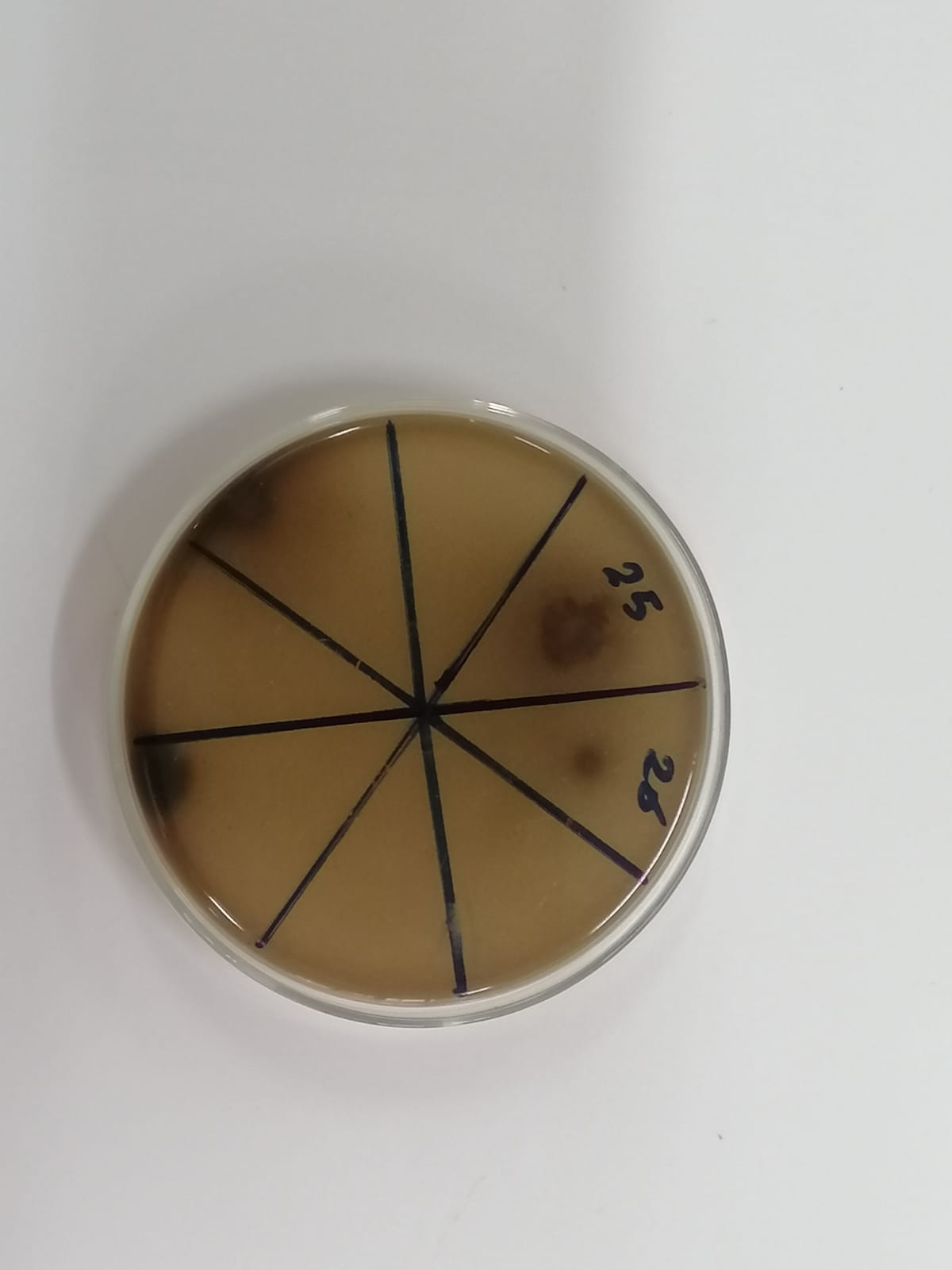


**S1. D**

**S2. Effect of phenolic compounds pyrogallol of the growth of 26 bacterial isolates**


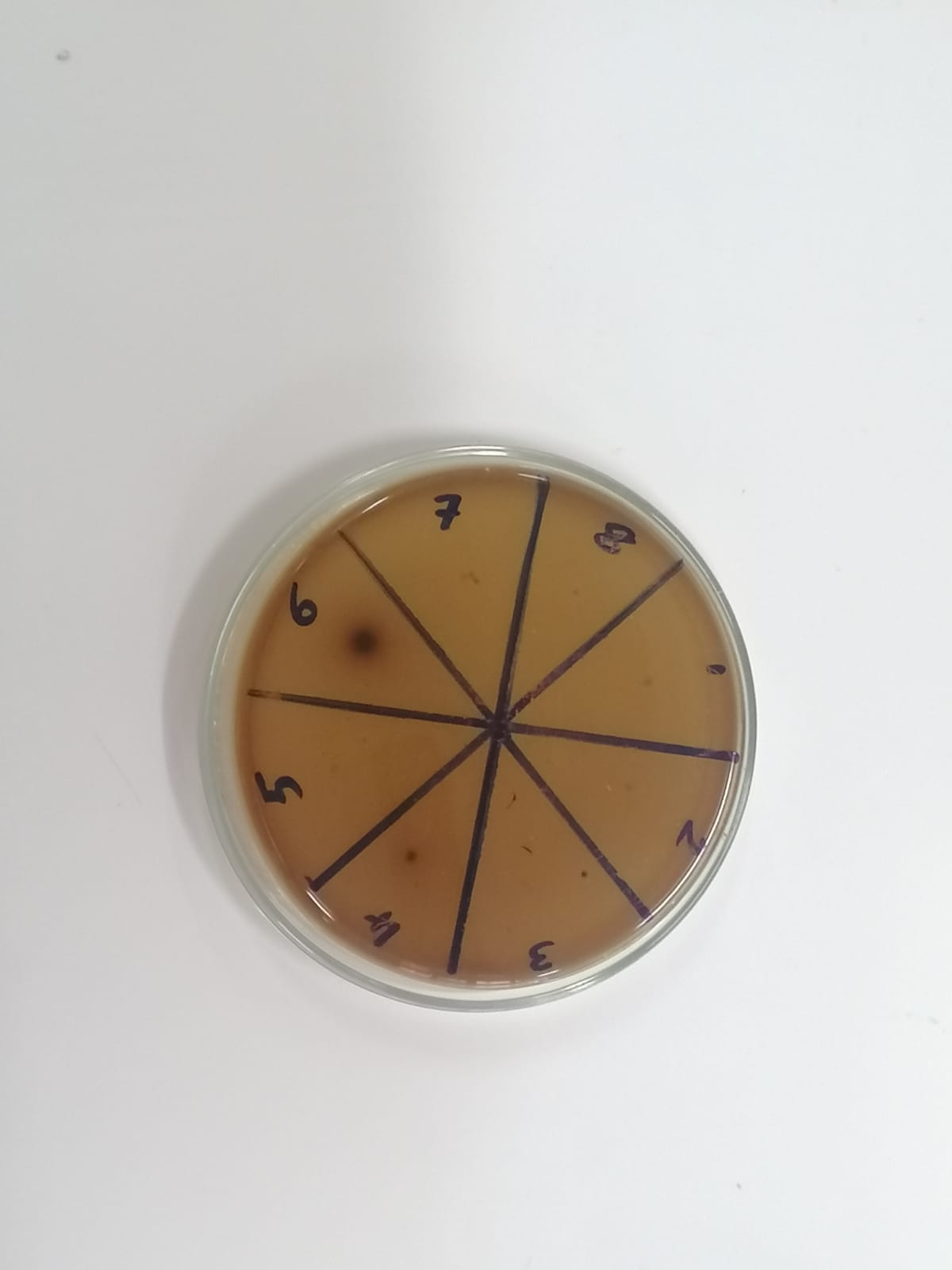

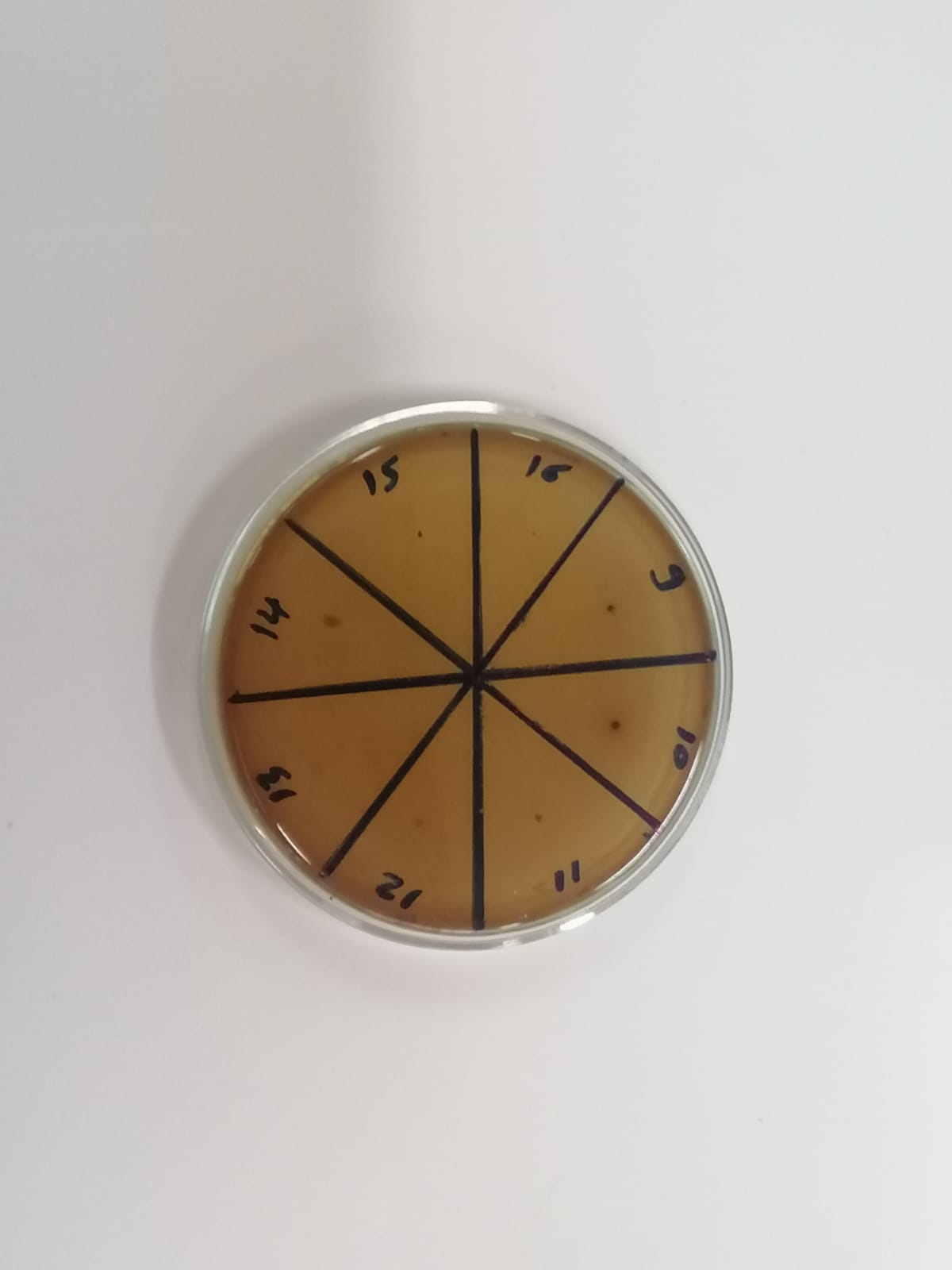

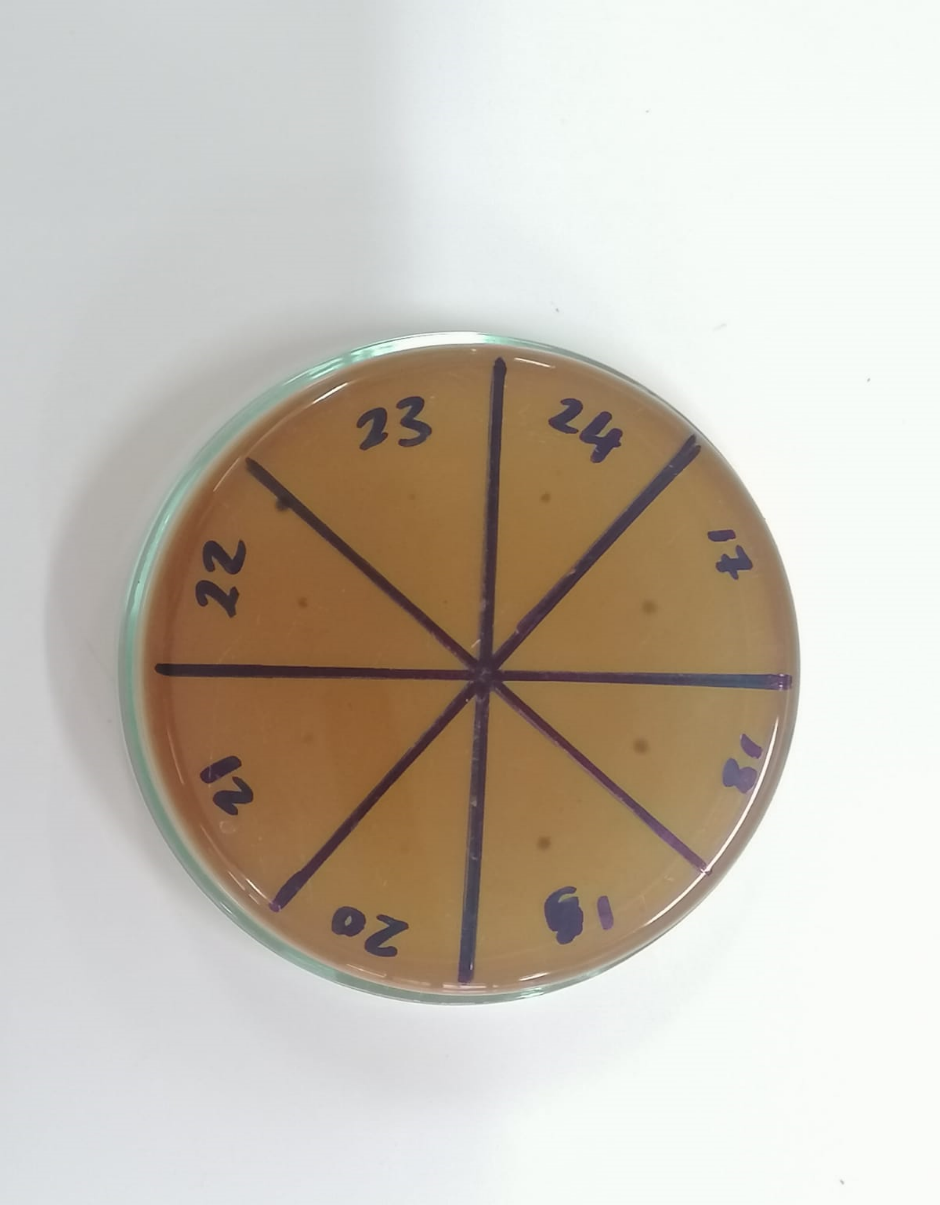

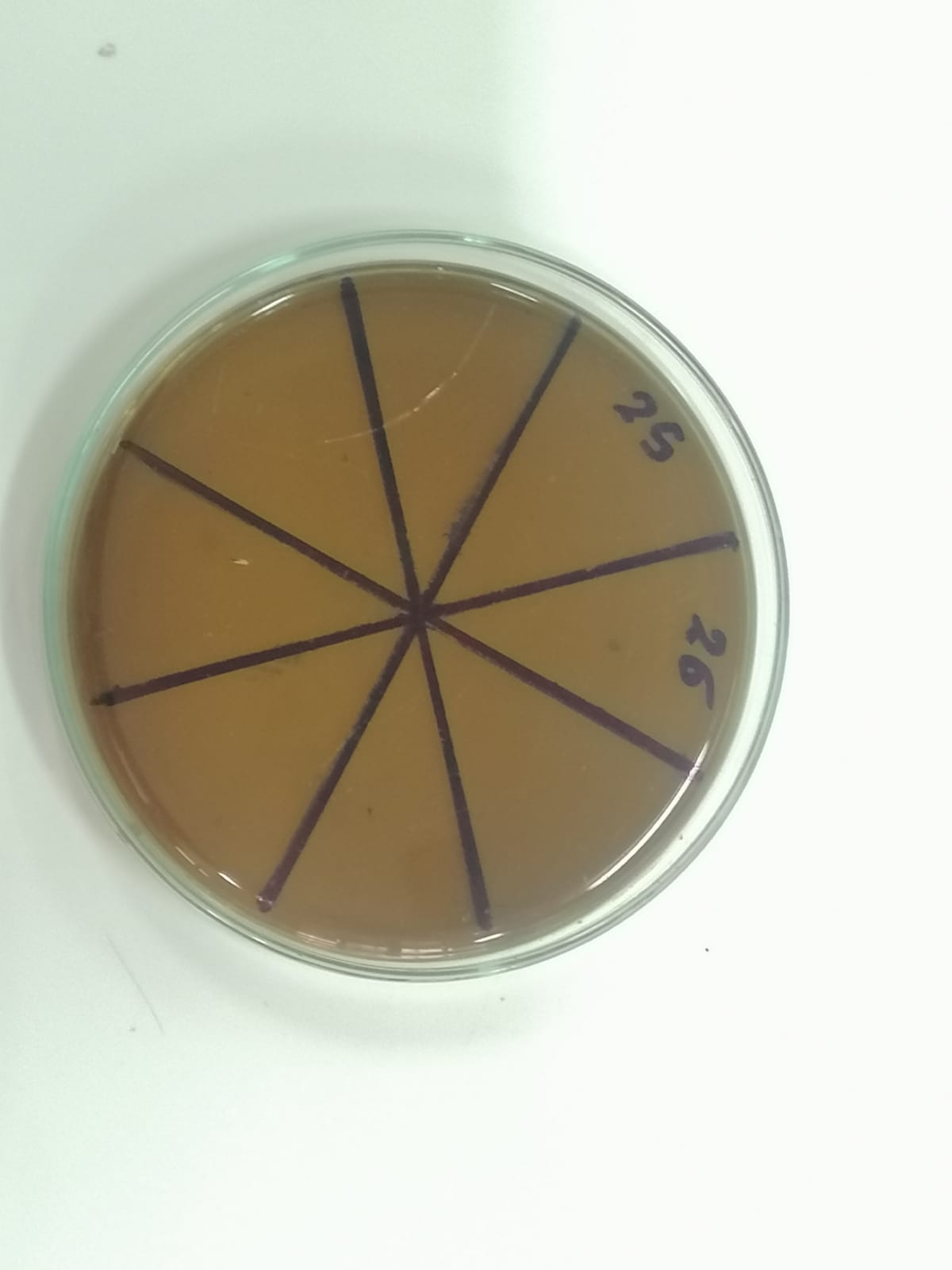

Supplement: Supplementary file 1 — Supplementary Material 1 [file 12896_2025_1080_MOESM1_ESM.docx]
